# Novel biomarkers for prediction of atonic postpartum hemorrhage among ‘low-risk’ women in labor

**DOI:** 10.3389/fimmu.2024.1416990

**Published:** 2024-07-11

**Authors:** Pei Zhang, Yanju Jia, Hui Song, Yifan Fan, Yan Lv, Hao Geng, Ying Zhao, Hongyan Cui, Xu Chen

**Affiliations:** ^1^ School of Medicine, Nankai University, Tianjin, China; ^2^ Tianjin Key Laboratory of Human Development and Reproductive Regulation, Tianjin, China; ^3^ Department of Obstetrics, Tianjin Central Hospital of Gynecology Obstetrics, Tianjin, China

**Keywords:** atonic postpartum hemorrhage, cytokine, hemogram, coagulation, prediction, biomarker

## Abstract

**Background:**

Postpartum hemorrhage (PPH) is the primary cause of maternal mortality globally, with uterine atony being the predominant contributing factor. However, accurate prediction of PPH in the general population remains challenging due to a lack of reliable biomarkers.

**Methods:**

Using retrospective cohort data, we quantified 48 cytokines in plasma samples from 40 women diagnosed with PPH caused by uterine atony. We also analyzed previously reported hemogram and coagulation parameters related to inflammatory response. The least absolute shrinkage and selection operator (LASSO) and logistic regression were applied to develop predictive models. Established models were further evaluated and temporally validated in a prospective cohort.

**Results:**

Fourteen factors showed significant differences between the two groups, among which IL2Rα, IL9, MIP1β, TNFβ, CTACK, prenatal Hb, Lymph%, PLR, and LnSII were selected by LASSO to construct predictive model A. Further, by logistic regression, model B was constructed using prenatal Hb, PLR, IL2Rα, and IL9. The area under the curve (AUC) values of model A in the training set, internal validation set, and temporal validation set were 0.846 (0.757–0.934), 0.846 (0.749–0.930), and 0.875 (0.789–0.961), respectively. And the corresponding AUC values for model B were 0.805 (0.709–0.901), 0.805 (0.701–0.894), and 0.901 (0.824–0.979). Decision curve analysis results showed that both nomograms had a high net benefit for predicting atonic PPH.

**Conclusion:**

We identified novel biomarkers and developed predictive models for atonic PPH in women undergoing “low-risk” vaginal delivery, providing immunological insights for further exploration of the mechanism underlying atonic PPH.

## Introduction

Postpartum hemorrhage (PPH), the leading cause of maternal mortality, affects 3%–10% of deliveries and accounts for nearly 27% of maternal deaths worldwide (1, 2). Researchers at numerous academic institutions have investigated related risk factors and developed tools to assess the risk of PPH, which merely stratified the risk of PPH based on clinical characteristics. However, emerging evidence suggests that these tools have limited predictive performance. For example, approximately 40% of women classified as high-risk individuals did not experience postpartum bleeding (3, 4). Although common risk factors increase the risk of PPH, our clinical data demonstrate that even low-risk women are susceptible to this condition. Additionally, the amount of bleeding is often underestimated and the importance of this symptom is overlooked, leading to delayed diagnosis and treatment and causing severe adverse outcomes among these women. The insufficiency in our current understanding of PPH highlights the need to explore novel indicators and establish a comprehensive predictive model.

Uterine atony continues to be the primary cause of PPH, accounting for approximately 70% of cases (5). Effective uterine contraction is crucial in reducing PPH, while factors that impede proper contraction can lead to atonic PPH and even death in severe cases. Almost all women giving birth are at risk of bleeding due to uterine atony. Compared to cesarean section, where timely measures can be taken, vaginal deliveries pose a greater risk to maternal and child safety due to the more sudden onset of PPH and limited access to emergency resources, especially in low-resource countries.

Recent studies have shown the crucial role of immune cells and cytokines in maintaining pregnancy and initiating labor (6, 7). Previous research found a correlation between certain cytokines and preterm birth, with some markers being able to predict delivery timing and adverse pregnancy outcomes (8, 9). The onset of labor is accompanied by substantial changes in fetomaternal physiology that facilitate successful delivery. These changes include the disruption of immune tolerance within the fetal membrane and placenta due to immune infiltration, as well as changes in the maternal internal immune environment (10, 11). Notably, evident chemotaxis and infiltration of neutrophils and macrophages into the myometrium occur after labor begins, leading to increased expression of inflammatory cytokines (12, 13). The secretion of cytokines plays a crucial role in the initiation and progression of uterine contractions and labor during pregnancy (14, 15). However, the association between maternal inflammatory cytokines in labor and uterine atony has not been investigated. Besides, recent studies have suggested that hemogram and coagulation parameters may also be related to this inflammatory response.

The predictive value of clinical features for uterine atony among low-risk women undergoing vaginal delivery is extremely limited. Currently, no clinical characteristics can accurately predict postpartum atony in the general population. Therefore, this study was initiated to analyze plasma cytokines in women undergoing vaginal delivery and establish a predictive model by integrating hemogram and coagulation parameters, providing valuable insights for identifying atonic PPH in the general population.

## Materials and methods

### Patients

Utilizing our extensive biobank, we conducted a nested case-control study to identify plasma biomarkers associated with atonic PPH in ‘low-risk’ primiparous women from April 2022 to November 2023. The inclusion criteria were as follows: primipara with full-term singleton infant, fetal head presentation, and age range of 18 to 34 years. The following exclusion criteria were applied: (1) women with autoimmune diseases; (2) women with pregnancy comorbidities and complications potentially associated with PPH (including preeclampsia, poorly controlled or medically controlled gestational diabetes mellitus, placental abruption, scarred uterus, coagulopathy, and induced labor); (3) women who delivered low-birth-weight infants or macrosomia clinically diagnosed after birth; (4) PPH cases caused by placental factors (identified through ultrasound and placental examination); (5) traumatic PPH cases with severe lacerations of the birth canal; (6) women with intrauterine infection; (7) cases positive for group B streptococcus. Given the high prevalence of obstetric lacerations, it is worth noting that most minor ones do not result in significant PPH. Therefore, our study included individuals with mild obstetric lacerations, defined as mild cervical lacerations (<1cm) or first-degree lacerations of perineum. Furthermore, these lacerations would be confirmed by a senior doctor and not considered as the primary cause of bleeding.

The blood samples were collected during the latent phase of the first stage of labor, which was defined as a period characterized by regular uterine contractions and progressive changes of the cervix, including some degree of effacement and slower progression of dilatation up to 5 cm (16). PPH was diagnosed based on excessive bleeding (≥ 500 ml) in the first 24 h after birth. This study employed volumetric methods instead of traditional approaches to accurately quantify blood loss (17). In brief, intrapartum blood loss was measured using a V-shaped blood collection bag with scale lines and warning lines, which allows the blood loss to be read at any time (**Supplementary Figure S1**). Atonic PPH was diagnosed based on specific criteria: vaginal bleeding (≥ 500 ml) following spontaneous placental expulsion and identification of a flaccid uterus during physical examination.

Eligible women who experienced atonic PPH after vaginal delivery were assigned to the atonic PPH group, while the remaining pregnant women without PPH were matched to the atonic PPH group in a 1:1 ratio using propensity scores. Gestational age, BMI (both before pregnancy and at delivery), and newborn birth weight were considered as predictors for PPH outcome during sample collection, and logistic regression was used to calculate the propensity score. Propensity score matching (PSM) was performed using maximized execution performance without replacement, with a caliper width set at 0.02 standard deviations of the logit of propensity score. Finally, a training cohort comprising 40 women diagnosed with atonic PPH and their matched controls was established. Similarly, in the prospective validation cohort, samples were collected from 41 women who experienced atonic PPH and matched with 41 normal controls.

**Figure 1:**
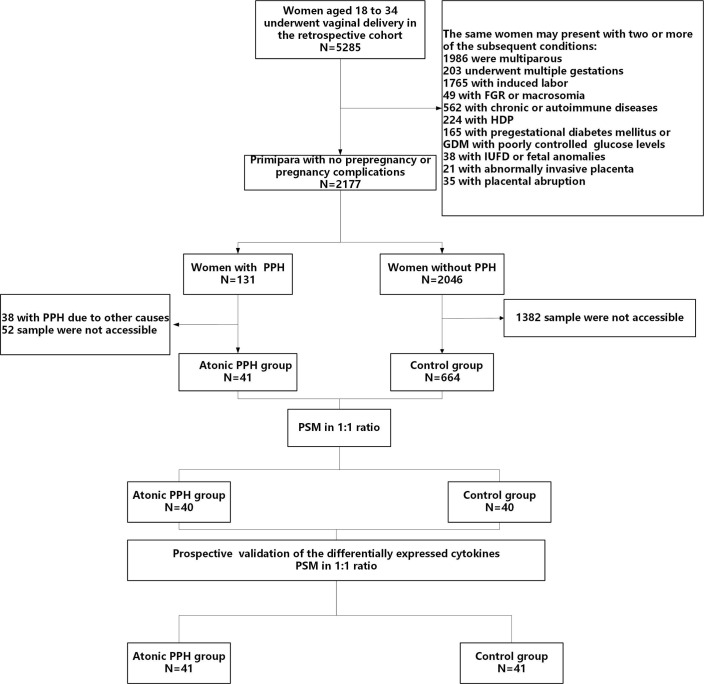
Flow diagram illustrating the process of participant screening and enrollment. *FGR*, fetal growth restriction; *HDP*, hypertensive disorders of pregnancy; *GDM*, gestational diabetes mellitus; *IUFD*, intrauterine fetal death; *PPH*, postpartum hemorrhage; *PSM*, propensity score matching.

The baseline characteristics were extracted from the electronic medical record, while data on obstetric and perinatal complications were collected through timely follow-up by trained nurses. Written informed consent was obtained from each subject, and this study was approved by the Ethical Review Committee of the local hospital (2022KY 063). The study flow is shown in **Figure 1**.

### Sample collection

Specimens were collected during the latent first stage after spontaneous labor. Samples of 6 ml of peripheral blood were taken from each participant via venipuncture using EDTA anticoagulant tubes for detection. After centrifugation at 3000 rpm for 10 min at 4°C, the supernatant plasma was separated and stored at −80°C.

### Assessment of potential biomarkers

#### Cytokine profiling

The frozen-thawed plasma was centrifuged and diluted 2-fold with a diluent. Subsequently, a 50 μL sample was taken for testing. The Bio-Plex Pro Human Cytokine Screening 48-Plex Panel (Bio-Rad, #12007283) was used to detect the levels of 48 cytokines (**Table 1**). The detailed procedures for biomarker measurement methods can be found in **Additional File 1**: **Detailed Methods**. Representative assay working ranges, assay sensitivity, and precision are presented in Additional Files (**Supplementary Table S1**).

**Table 1:** Comparisons of concentrations of cytokines between the atonic PPH and matched control group in training cohort.

Analyte (pg/ml)	Control N=40	Atonic PPH N=40	*P* value	Analyte (pg/ml)	Control N=40	Atonic PPH N=40	*P* value
Basic FGF	13.09 (9.65, 19.21)	9.65 (8.73, 13.09)	0.092	IL-12(p70)	1.21 (1.05, 1.38)	1.21 (1.05, 1.38)	1.000
CTACK	61.91 (54.95, 82.50)	78.57 (60.26, 101.37)	0.033	IL-13	2.52 (1.71, 3.82)	2.64 (1.65, 4.80)	0.847
Eotaxin	28.35 (24.09, 36.96)	26.27 (21.62, 31.93)	0.329	IL-16	21.28 (17.9, 40.35)	24.26 (15.71, 53.11)	0.769
G-CSF	79.63 (73.02, 92.67)	76.25 (64.12, 92.67)	0.481	IL-17	3.70 (3.70, 4.58)	3.91 (3.30, 4.13)	0.755
GM-CSF	0.11 (0.08, 0.17)	0.12 (0.08, 0.21)	0.403	IL-18	28.04 (12.88, 79.43)	19.83 (6.65, 97.72)	0.436
GRO-α	229.02 (138.97, 489.15)	193.08 (61.76, 323.89)	0.053	IP-10	322.74 (230.69, 411.78)	350.08 (261.29, 441.80)	0.341
HGF	208.36 (191.07, 237.27)	227.21 (185.00, 280.65)	0.214	LIF	23.02 (18.46, 36.84)	27.57 (18.46, 42.16)	0.693
TRAIL	2.42 (1.19, 5.05)	1.69 (1.19, 3.45)	0.156	MCP-1	5.29 (3.80, 6.86)	5.07 (3.12, 8.52)	0.827
IFN-α2	2.99 (2.44, 4.44)	3.50 (2.26, 4.27)	0.938	MCP-3	0.36 (0.25, 0.41)	0.36 (0.25, 0.36)	0.778
IFN-γ	2.91 (1.86, 4.60)	2.32 (1.65, 4.80)	0.460	M-CSF	15.68 (10.05, 21.70)	15.34 (10.17, 23.54)	0.992
IL-1α	7.72 (7.72, 10.55)	9.14 (7.23, 10.55)	0.949	MIF	391.61 (298.70, 483.86)	387.37 (298.70, 502.36)	0.773
IL-1β	1.75 (0.98, 3.30)	1.75 (1.13, 2.57)	0.992	MIG	44.29 (30.98, 61.96)	42.00 (26.34, 61.30)	0.274
IL-1ra	158.57 (125.04, 234.19)	158.57 (114.66, 210.15)	0.846	MIP-1a	0.98 (0.86, 1.19)	0.96 (0.71, 1.19)	0.170
IL-2	0.91 (0.75, 1.47)	1.09 (0.65, 1.30)	0.827	MIP-1β	203.54 (197.02, 219.56)	192.80 (176.80, 209.12)	0.008
IL-2Rα	26.76 (22.21, 36.16)	20.14 (15.53, 29.14)	0.001	β-NGF	0.24 (0.18, 0.42)	0.25 (0.18, 0.41)	0.900
IL-4	1.46 (1.32, 2.17)	1.46 (1.12, 2.11)	0.464	PDGF-BB	38.10 (15.68, 130.42)	16.08 (6.64, 151.99)	0.100
IL-6	0.02 (0.01, 0.17)	0.01 (0.00, 0.72)	0.918	RANTES	1944.00 (1667.25, 2159.00)	1966.00 (1424.75, 2378.00)	0.711
IL-7	0.66 (0.40, 1.16)	0.70 (0.40, 1.24)	0.904	SCF	105.11 (88.39, 130.53)	96.39 (81.05, 123.68)	0.148
IL-8	2.00 (1.17, 3.97)	1.69 (0.98, 3.51)	0.700	SCGF-β	12796.50 (9655.00, 15810.50)	11734.00 (9948.50, 13508.00)	0.303
IL-9	168.71 (161.56, 180.04)	154.71 (148.15, 179.08)	0.006	SDF-1α	679.77 (606.69, 757.62)	698.40 (630.10, 853.32)	0.161
IL-10	2.72 (1.55, 4.07)	350.08 (261.29, 441.80)	0.977	TNF-α	18.48 (15.85, 21.51)	17.58 (14.83, 19.92)	0.193
IL-12(p40)	39.38 (39.38, 64.16)	41.28 (28.85, 64.16)	0.483	TNF-β	163.48 (147.50, 178.27)	151.55 (131.64, 170.72)	0.015

The results are reported as the median and interquartile range (25th to 75th percentiles) of measured cytokine concentrations (pg/ml).

Cytokines such as IL3, VEGF-A, IL5, and IL15 that remained undetectable during pregnancy were excluded from the analysis.

Basic FGF, basic fibroblast growth factor; CTACK, cutaneous T cell-attracting chemokine; G-CSF, granulocyte-colony stimulating factor; GM-CSF, granulocyte-macrophage colony stimulating factor; GRO-α, growth-regulated oncogene-α; HGF, hepatocyte growth factor; IFN-α2, interferon-α2; IFN-γ, interferon-γ; IL, interleukin; IP-10, interferon-inducible protein-10; LIF, leukemia inhibitory factor; MCP-1, monocyte chemotactic protein-1; MCP-3, monocyte chemotactic protein-3; M-CSF, macrophage colony stimulating factor; MIF, migration inhibitor factor; MIG, monokine induced by IFN-γ; MIP-1α, macrophage inflammatory protein-1α; MIP-1β, macrophage inflammatory protein-1β; β-NGF, β-nerve growth factor; PDGF-BB, platelet-derived growth factor-BB; RANTES, regulate upon activation normal T cell expressed and secreted; SCF, stem cell factor; SCGF-β, stem cell growth factor-β; SDF-1α, stromal cell-derived factor-α; TNF-α, tumor necrosis factor-α; TNF-β, tumor necrosis factor-β; TRAIL, tumor necrosis factor-related apoptosis-induced ligand; VEGF-A, vascular endothelial growth factor A; VCAM-1, vascular cell adhesion molecule-1.

#### Routine laboratory tests

Results of the last laboratory test before delivery, including whole blood cell analysis and coagulation tests, were obtained upon patients’ admission. The complete blood count parameters were analyzed using Cell-Dyn 3700 (Abbott Diagnostics, Santa Clara, CA, USA). Coagulation tests were conducted using the Sysmex CS 2500 System coagulation analyzer (Siemens Healthcare Diagnostics, Erlangen, Germany). All measurements were performed within a 2-h time frame after blood sampling. Neutrophil-to-lymphocyte ratio (NLR), monocyte-to-lymphocyte ratio (MLR), platelet-to-lymphocyte ratio (PLR), systemic immune-inflammation index (SII), and LnSII were calculated based on these laboratory test parameters, which were previously reported to be related to systemic immune response. The SII was obtained by multiplying the neutrophil count by the PLR and LnSII was the natural logarithm of SII.

#### Enzyme-linked immunosorbent assay (ELISA)

The concentrations of differentially expressed cytokines were verified in the temporal validation cohort. Human IL2Rα, IL9, MIP1β, TNFβ, and CTACK ELISA kits from Thermo Fisher Scientific (Waltham, MA, USA) were employed for prospective temporal validation. Plasma samples were appropriately diluted with manufacturer-provided diluents to ensure that absorbance readings fell within the range of the standard curve. The concentration of each sample was determined by constructing a linear standard curve using the provided software, in accordance with the manufacturer’s instructions.

### Statistical analysis

Statistical analysis was performed using SPSS 26.0 (IBM, Armonk, NY, USA), R 4.3.1 (R Foundation for Statistical Computing, Vienna, Austria), and GraphPad Prism 9.0 (GraphPad Software, San Diego, CA, USA). For continuous variables, normally distributed data are presented as mean ± standard deviation, and non-normally distributed data are presented as median and interquartile range. Independent samples t-test was employed for normally distributed continuous variables, whereas Mann-Whitney U test was used for non-normally distributed ones. Paired comparisons were conducted using the Wilcoxon paired test. Categorical variables are presented as counts (percentages) and were compared using chi-square analysis or Fisher’s exact test. Statistical significance in cytokines was determined at *P*< 0.05. Multiple comparisons in hemogram and coagulation parameters were adjusted for using a false discovery rate (FDR)<0.1. Differentially expressed cytokines, hemogram, and coagulation parameters were incorporated into subsequent variable screening and the construction of predictive models.

Considering the limited number of observed events, we used least absolute shrinkage and selection operator (LASSO) regression with tenfold cross-validation to mitigate potential collinearity and overfitting issues. A higher *λ* value would shrink smaller coefficients towards zero, retaining only the most influential predictors. To ensure model conciseness, we selected the *λ*_min_ resulting in nine predictors to construct predictive model A. Then, we further analyzed significant variables selected by LASSO regression using logistic regression to identify predictors to construct model B. Risk assessment nomograms for atonic PPH were generated. The models established using the training set were validated. Internal validation was performed using the bootstrap method (1000 times), while temporal validation used prospective cohort data. Discrimination of the model was evaluated by drawing a receiver operating characteristic curve (ROC) and calculating the area under the curve (AUC). Calibration curves were plotted to visualize the calibration of the nomograms and assessed using Hosmer–Lemeshow (H-L) chi-square statistics. Additionally, the clinical utility of the predictive models was assessed by conducting decision curve analyses (DCA) to demonstrate the potential net benefit of the model at various threshold probabilities.

## Results

### Baseline characteristics and pregnancy outcomes

In the retrospective cohort, a total of 5285 women underwent vaginal deliveries. After applying the predetermined inclusion and exclusion criteria and considering the availability of blood samples, we included 40 ‘low-risk’ women with atonic PPH and matched them with 40 normal controls. For temporal validation, we prospective recruited 41 women who developed atonic PPH and matched them with 41 normal controls (**Figure 1**). The baseline characteristics, including maternal age, BMI, blood pressure, gestational age, newborn birth weight, and other characteristics were comparable in both two pairs of compared groups (**Table 2**).

**Table 2:** Baseline characteristics of participants.

Characteristics	Training cohort		*P* value	Temporal validation cohort		*P* value
	Control (n=40)	Atonic PPH (n=40)		Control (n=41)	Atonic PPH (n=41)	
Maternal age (years)	28.45 ± 2.76	28.93 ± 2.90	0.46	28.73 ± 3.23	29.20 ± 3.04	0.51
BMI before pregnancy (kg/m^2^)	21.43 ± 1.85	21.44 ± 1.72	0.97	21.32 ± 2.56	21.23 ± 2.52	0.89
BMI at delivery (kg/m^2^)	27.49 ± 3.11	27.49 ± 3.00	0.99	26.56 ± 2.83	26.57 ± 2.83	0.98
Gestational age (week)	39.31 ± 0.98	39.36 ± 0.85	0.77	39.64 ± 0.75	39.69 ± 0.94	0.78
ART no./total no. (%)	3/40 (7.5)	3/40 (7.5)	1.00	1/41 (2.4)	2/41 (4.9)	0.56
GDM no./total no. (%) **^a^**	6/40 (15.0)	7/40 (17.5)	0.76	5/41 (12.2)	5/41 (12.2)	1.00
Newborn birth weight (g)	3430.00 ± 380.03	3432.25 ± 344.01	0.98	3458.29 ± 389.28	3359.02 ± 556.39	0.35
1-min Apgar score	10.00 (10.00, 10.00)	10.00 (10.00, 10.00)	0.56	10.00 (10.00, 10.00)	10.00 (10.00, 10.00)	1.00
5-min Apgar score	10.00 (10.00, 10.00)	10.00 (10.00, 10.00)	1.00	10.00 (10.00, 10.00)	10.00 (10.00, 10.00)	1.00
5-minute Apgar score< 7 no./total no. (%)	0/40 (0)	0/40 (0)	–	0/40 (0)	0/40 (0)	–
Episiotomy no./total no. (%)	10/40 (25.0)	12/40 (30.0)	0.617	10/31 (24.4)	14/41 (29.3)	0.332
Mild obstetric lacerations no./total no. (%) **^b^**	25/40 (62.5)	23/40 (57.5)	0.648	24/41 (58.5)	26/41 (63.4)	0.651
Blood loss at bag removal (ml) **^c^**	286.00 ± 64.44	712.38 ± 200.97	<0.001	282.68 ± 68.89	821.83 ± 308.99	<0.001
Blood loss within 24 h after delivery (ml) **^d^**	366.75 ± 62.55	818.35 ± 206.55	<0.001	365.98 ± 67.36	951.20 ± 298.55	<0.001

Continuous variables are reported as mean ± standard deviation or median (interquartile range), while categorical variables are presented as number (percentage). BMI, Body mass index; GDM, gestational diabetes mellitus; PPH, postpartum hemorrhage; ART, assisted reproductive technology.

**^a^** GDM cases refer to women who can manage their blood glucose levels through diet and exercise, without medication. This involves achieving fasting blood glucose level < 5.3 mmol/L (95 ml/dl), postprandial 2-hour blood glucose level < 6.7 mmol/L, and maintaining HbA1c levels within a controlled range of 6% (42 mmol/mol).

**^b^** Mild obstetric lacerations are defined as first-degree perineal lacerations or cervical lacerations (<1cm). The included.

**^c^** The intrapartum blood loss was collected in a graduated collection bag, which was subsequently removed when the patient left the delivery room.

**^d^** The total blood loss within 24 hours was calculated by summing the intrapartum blood loss (collected in a bag) and the postpartum blood loss in the ward (measured by weight).

### Analysis of maternal circulating cytokines revealed dysregulation in five cytokines associated with atonic PPH

**Figure 2:**
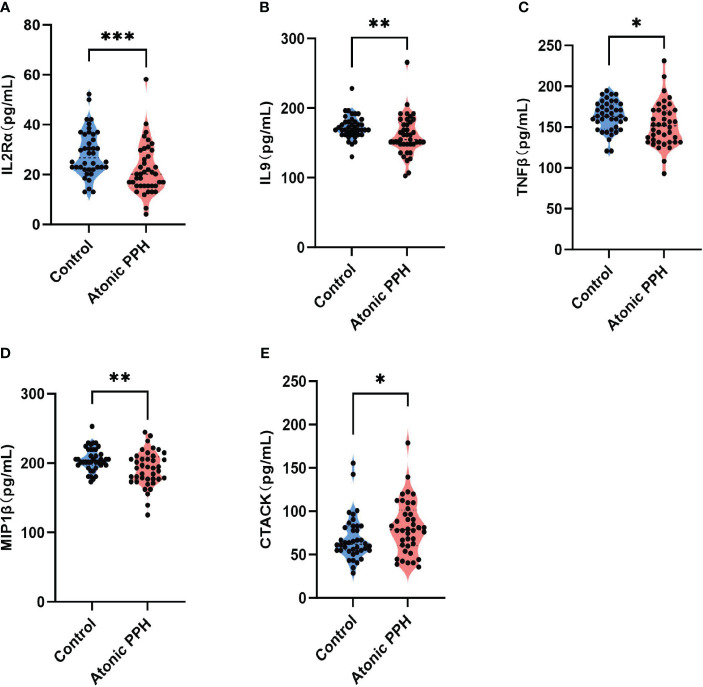
**(A–E)** Violin plots show significant differences in 5 plasma cytokines between the atonic PPH group and the normal control group, including **(A)** *IL-2rα*, **(B)** *IL-9*, **(C)** *TNF-β*, **(D)** *MIP1β* and **(E)** *CTACK*. *IL-2rα*, interleukin−2Rα; *IL-9*, interleukin−9; *TNF-β*, tumor necrosis factor−β; *MIP1β*, macrophage inflammatory protein-1β*; CTACK*, cutaneous T cell-attracting chemokine. Mann-Whitney U test was used for assessing intergroup differences across these factors. * P<0.05; ** P<0.01; *** P<0.001.

**Table 1** provides a summary of the cytokine expression profile in both the atonic PPH and control groups. And to compare differences in cytokine levels across different groups, overall cytokine data was visualized in a heat map representation (**Supplementary Figure S2**). Besides, pairwise Pearson’s correlation analysis between cytokines was performed in the training cohort (**Supplementary Figure S3**). Among these cytokines, the expression levels of IL2Rα, IL9, TNFβ, and MIP1β in the PPH group were significantly lower than those in the control group. Conversely, CTACK exhibited significantly higher levels in atonic PPH than in the control group. Violin plots were generated for these five cytokines (**Figure 2**).

### Analysis of hemogram and coagulation parameters identified nine biomarkers associated with atonic PPH

**Figure 3:**
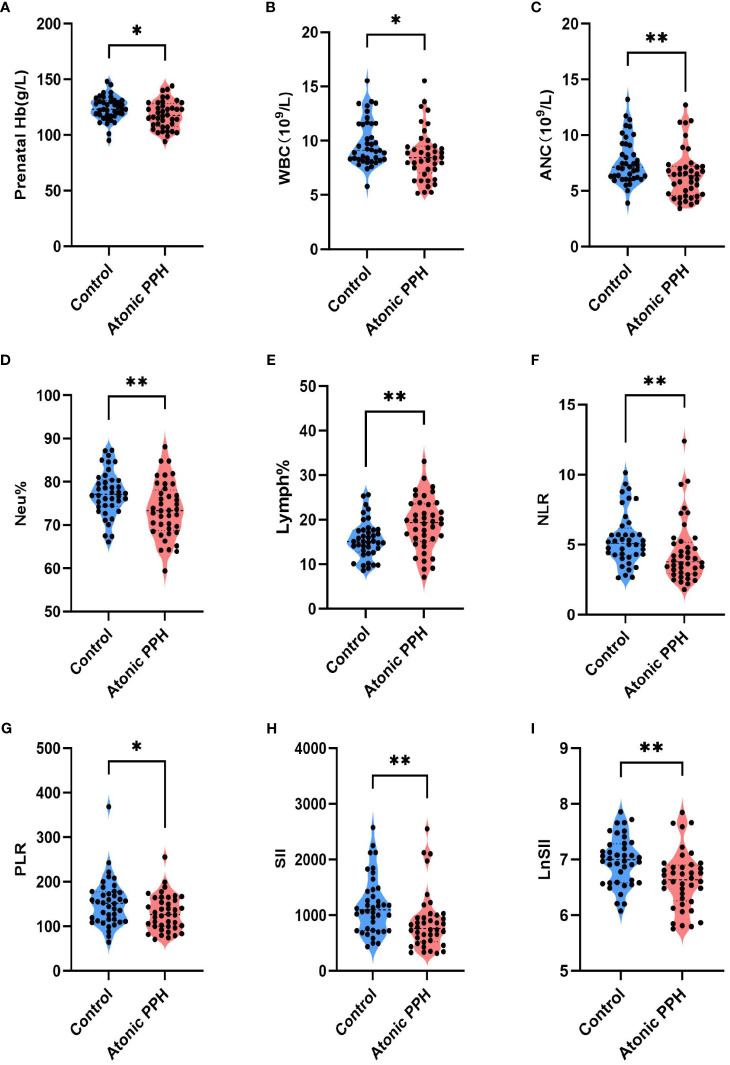
**(A–I)** Violin plots show significant differences in 9 hemogram and coagulation indicators between the atonic PPH group and the normal control group, including **(A)** *Prenatal Hb*, **(B)** *WBC*, **(C)** *ANC*, **(D)** *Neu%*, **(E)** *Lymph%*, **(F)** *NLR*, **(G)** *PLR*, **(H)** *SII* and **(I)** *LnSII*. *Hb*, hemoglobin; *WBC*, white blood cell count; *ANC*, absolute neutrophil count; *Neu%*, proportion of neutrophils; *Lymph%*, proportion of lymphocytes; *NLR*, neutrophil-to-lymphocyte ratio; *PLR*, platelet-to-lymphocyte ratio; *SII*, systemic immune-inflammation index; *LnSII*, natural logarithm of SII. Independent samples t-test was employed for *Prenatal Hb, Neu%, Lymph% and LnSII*. Mann-Whitney U test was used for *WBC, ANC, NLR, PLR*, and *SII*. * P<0.05; ** P<0.01; *** P<0.001.

Peripheral blood cell parameters and coagulation parameters in the two groups were compared; detailed results are provided in **Table 3**. The findings revealed that prenatal Hb (P = 0.032, adjusted P = 0.064), WBC (P = 0.020, adjusted P = 0.057), ANC (P = 0.008, adjusted P = 0.027), Neu% (P = 0.005, adjusted P = 0.020), NLR (P = 0.004, adjusted P = 0.020), PLR (P = 0.030, adjusted P = 0.064), SII (P = 0.002, adjusted P = 0.020) and LnSII (P = 0.002, adjusted P = 0.020) were significant lower in the atonic PPH group than that in the control group. Conversely, women in the atonic PPH group had a higher level of Lymph% (P = 0.004, adjusted P = 0.020) and D-dimer (P = 0.028, adjusted P = 0.064) compared to matched controls (**Table 3**). Violin plots depicting these nine biomarkers were generated (**Figure 3**).

**Table 3:** Hemogram and coagulation indices in atonic PPH and matched controls in training cohort.

Parameters	Control N=40	Atonic PPH N=40	*P* value	*Adjusted P* value
Prenatal Hb (pg/ml)	123.78 ± 10.78	118.10 ± 12.35	0.032	0.064
WBC (10^9^/L)	9.13 (8.28, 11.57)	8.44 (7.03,9.50)	0.020	0.057
ANC (10^9^/L)	7.12 (6.13, 9.18)	6.34 (4.58,7.18)	0.008	0.027
Neu%	77.31 ± 5.26	73.53 ± 6.45	0.005	0.020
LYC (10^9^/L)	1.48 ± 0.32	1.55 ± 0.37	0.334	0.393
Lymph%	15.59 ± 4.28	19.02 ± 5.91	0.004	0.020
MONO (10^9^/L)	0.61 ± 0.20	0.58 ± 0.18	0.483	0.537
MONO %	6.26 ± 1.88	6.65 ± 1.35	0.295	0.373
PLT (10^9^/L)	208.50 (176.50, 234.75)	183.00 (148.25,236.25)	0.104	0.160
NLR	5.10 (4.27, 6.18)	3.78 (2.93, 5.19)	0.004	0.020
MLR	0.39 (0.31, 0.51)	0.35 (0.28,0.45)	0.254	0.363
PLR	151.80 (113.25,177.75)	126.48 (99.82, 162.95)	0.030	0.064
SII	1095.69 (723.89,1452.64)	761.37 (523.14,989.44)	0.002	0.020
LnSII	6.98 ± 0.44	6.64 ± 0.52	0.002	0.020
PT (s)	10.74 ± 0.49	10.96 ± 0.67	0.093	0.155
APTT (s)	25.18 ± 2.26	25.34 ± 1.91	0.738	0.738
TT (s)	16.60 (16.13,17.18)	16.3 (15.8,17.3)	0.573	0.603
INR	0.91 ± 0.04	0.94 ± 0.06	0.066	0.120
Fib (g/L)	4.36 (4.03,4.78)	4.19 (3.59,4.94)	0.298	0.373
D-D (mg/L)	1.61 (1.09, 2.44)	2.17 (1.45,3.57)	0.028	0.064

Data are presented as mean ± standard deviation or median (interquartile range). The P value was adjusted using the Benjamini–Hochberg procedure to control the false discovery rate (FDR q-value) for multiple testing.

Hb, hemoglobin; Hct, hematocrit; WBC, white blood cell count; ANC, absolute neutrophil count; Neu%, neutrophil ratio; LYC, lymphocyte count; Lymph%, lymphocyte ratio; MONO, monocyte count; MONO%, monocyte ratio; PLT, platelet count; NLR, neutrophil to lymphocyte ratio; MLR, monocyte to lymphocyte ratio; PLR, platelet to lymphocyte ratio; SII, systemic immune-inflammation index; LnSII, natural logarithm of SII; PT, prothrombin time; APTT, activated partial thromboplastin time; TT, thrombin time; INR, international normalized ratio; Fib, fibrinogen; D-D, D-dimer.

### Screening of predictors for atonic PPH in low-risk women

**Figure 4:**
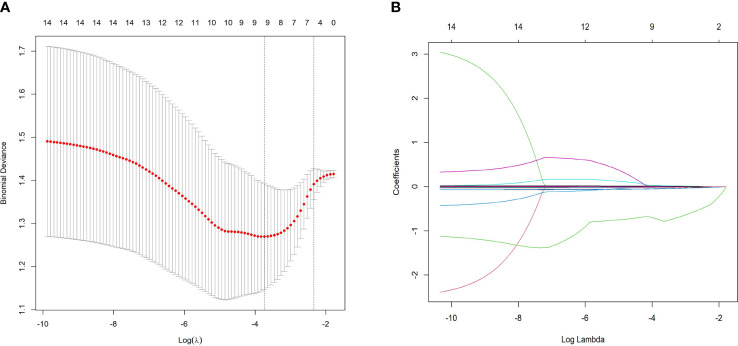
Predictor selection using LASSO regression analysis with tenfold cross-validation. **(A)** LASSO coefficient profiles of the 14 risk factors were created against the log (*λ*) sequence. **(B)** Tuning parameter (lambda, *λ*) selection of deviance in the LASSO regression based on the minimum criterion (left dotted line) and the 1-SE criterion (right dotted line). In the present study, predictor selection was performed according to the minimum criterion (including *Prenatal Hb, Lymph%, PLR, LnSII, IL2Rα, MIP1β, TNFβ, CTACK*, and *IL9*). *LASSO* least absolute shrinkage and selection operator, *SE* standard error.

**Figure 5:**
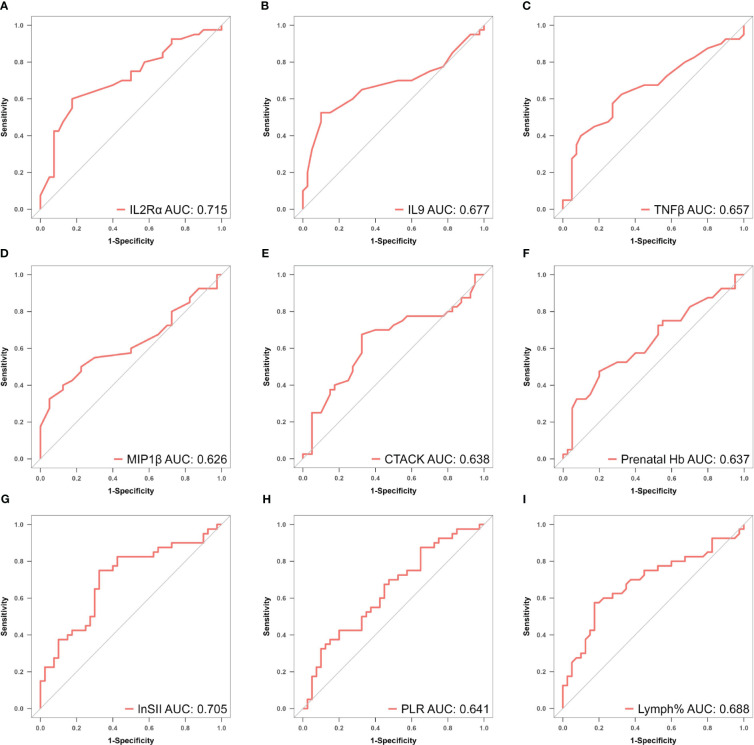
**(A–I)** ROC curves of identified biomarkers for atonic PPH: **(A)** IL−2Rα (AUC: 715, 95% CI 0.600–0.829, *P* < 0.001), **(B)** IL−9 (AUC: 0.677, 95% CI 0.554–0.800, *P* = 0.003), **(C)** TNF−β (AUC: 0.657, 95% CI 0.535–0.764, *P* = 0.017), **(D)** MIP1β(AUC: 0.626, 95% CI 0.501–0.752, *P* = 0.026), **(E)** CTACK (AUC: 0.638, 95% CI 0.513–0.767, *P* = 0.039), **(F)** Prenatal Hb (AUC: 0.637, 95% CI 0.514–0.760, *P* = 0.017), **(G)** Lymph% (AUC: 0.688, 95% CI 0.568–0.807, *P* = 0.002), **(H)** PLR (AUC: 0.641, 95% CI 0.519–0.762, *P* = 0.015), and **(I)** LnSII (AUC: 0.705, 95% CI 0.589–0.821, *P* = 0.001). *IL2Rα*, interleukin-2 receptor subunit α; *MIP1β*, macrophage inflammatory protein-1β; *TNF-β*, tumor necrosis factor-β; *IL9*, interleukin-9; *CTACK*, cutaneous T cell-attracting chemokine; *Hb*, hemoglobin; *Lymph%*, lymphocyte ratio; *PLR*, platelet-to-lymphocyte ratio; *SII*, systemic immune-inflammation index; *LnSII*, natural logarithm of SII; ROC, receiver operating characteristic; AUC, area under the ROC curve.

To mitigate the influence of multicollinearity between variables, LASSO was conducted for screening from the initial 14 differentially expressed variables (5 cytokines and 9 hemogram- and coagulation-related parameters). By gradually compressing the variable coefficients as the penalty coefficient *λ* changed (**Figure 4**), overfitting was avoided. When *λ*_min_=0.02388906, nine factors with non-zero regression coefficients were selected: IL−2Rα (AUC: 0.715, 95% CI 0.600–0.829, P< 0.001), IL−9 (AUC: 0.677, 95% CI 0.554–0.800, P = 0.003), TNF−β (AUC: 0.657, 95% CI 0.535–0.764, P = 0.017), MIP1β (AUC: 0.626, 95% CI 0.501–0.752, P = 0.026), CTACK (AUC: 0.638, 95% CI 0.513–0.767, P = 0.039), prenatal Hb (AUC: 0.637, 95% CI 0.514–0.760, P = 0.017), Lymph% (AUC: 0.688, 95% CI 0.568–0.807, P = 0.002), PLR (AUC: 0.641, 95% CI 0.519–0.762, P = 0.015), and LnSII (AUC: 0.705, 95% CI 0.589–0.821, P = 0.001) (**Supplementary Table S2**). Their ROC curves were presented in **Figure 5**.

### Construction of the predictive models

The nine factors selected by LASSO were all used to construct predictive model A (**Supplementary Table S3**). Subsequently, to further control the influence of confounding factors, the above nine factors were analyzed using logistic regression. Finally, only prenatal Hb, PLR, IL2Rα, and IL9 were determined to be independent predictors (*P*< 0.05), as shown in **Supplementary Table S4**. These four factors were used to develop model B accordingly. The formulas for both predictive models are presented below:


$$\begin{array}{c} \text{Model A log }\left(\frac{p}{1-p}\right)\\ \;=19.829-0.072\times Prenatal\;Hb+0.054\times Lymph\%-0.006\\ \times PLR-0.403\times lnSII-0.071\times IL2R\alpha -0.017\times MIP1\beta \\ -0.001\times TNF\beta +0.017\times CTACK-0.026\times IL9 \end{array}$$

$$\begin{array}{c} \text{Model B log }\left(\frac{p}{1-p}\right)\\ \;=18.986-0.078\times Prenatal\;Hb-0.014\times PLR-0.069\\ \times IL2R\alpha -0.025\times IL9 \end{array}$$

### Predictive nomograms for atonic PPH

**Figure 6:**
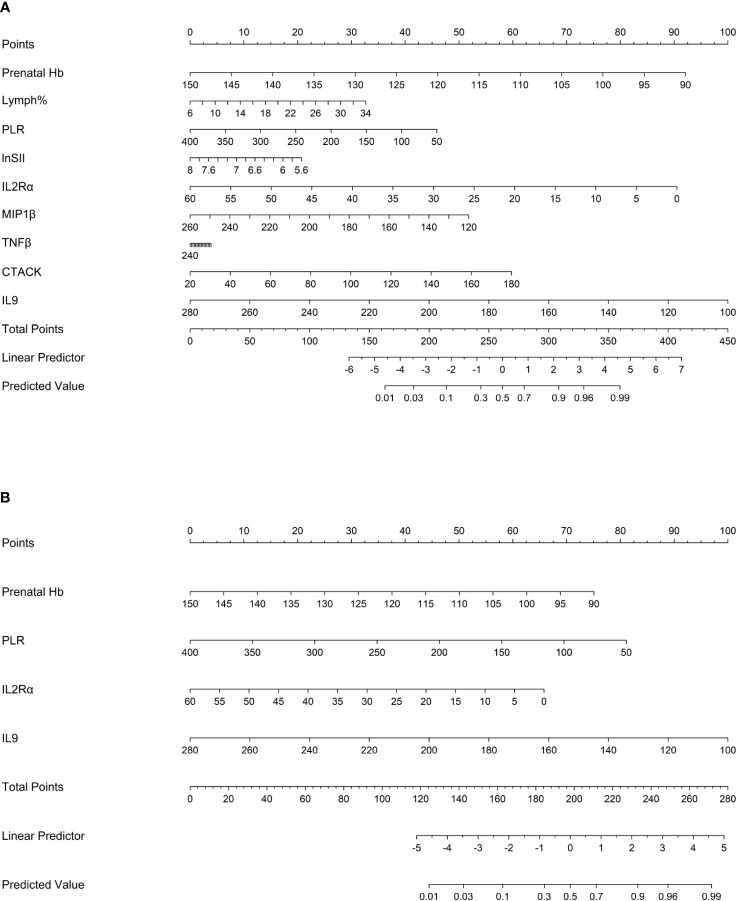
Nomogram1 and nomogram2 were constructed to predict the incidence of atonic PPH among women in labor. **(A)** Nomogram1 including IL2Rα, IL9, MIP1β, TNF-β, CTACK, Prenatal Hb, Lymph%, PLR, and LnSII for assessing the risk of atonic PPH among women in labor. **(B)** Nomogram2 including IL2Rα, IL9, Prenatal Hb, and PLR for assessing the risk of atonic PPH among women in labor. Nomogram1 and nomogram2 are used to obtain the risk of atonic PPH by adding up the points identified on the points’ scale for each variable. *IL2Rα*, interleukin-2 receptor subunit α; *MIP1β*, macrophage inflammatory protein-1β; *TNF-β*, tumor necrosis factor-β; *IL9*, interleukin-9; *CTACK*, cutaneous T cell-attracting chemokine; *Hb*, hemoglobin; *Lymph%*, lymphocyte ratio; *PLR*, platelet-to-lymphocyte ratio; *SII*, systemic immune-inflammation index; *LnSII*, natural logarithm of SII.

The “rms” package was used to draw nomograms for atonic PPH in women with vaginal delivery (**Figure 6**), which could predict the risk of atonic PPH in individual patients. In clinical application, the individual score for each predictor could be acquired and then added together to obtain the total score. Finally, on the number axis of the total score, the corresponding predicted probability projected downward would be the risk of atonic PPH for individuals.

### Temporal validation

**Figure 7:**
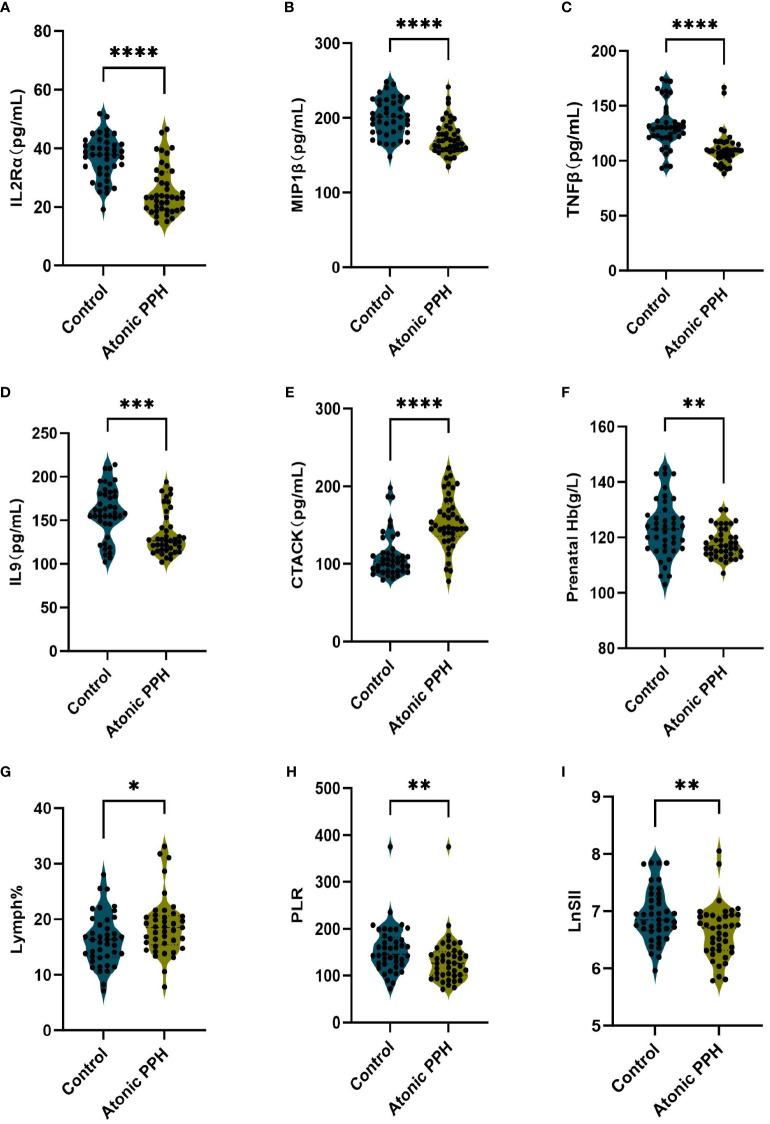
**(A–I)** Violin plots illustrate validated differentially expressed biomarkers between the atonic PPH group and control group in a prospective cohort, including **(A)** *IL-2rα*, **(B)** *IL-9*, **(C)** *TNF-β*, **(D)** *MIP1β*, **(E)** *CTACK*, **(F)** *Prenatal Hb*, **(G)** *Lymph%*, **(H)** *PLR* and **(I)** *LnSII. IL-2rα*, interleukin−2Rα; *IL-9*, interleukin−9; *TNF-β*, tumor necrosis factor−β; *MIP1β*, macrophage inflammatory protein-1β; *CTACK*, cutaneous T cell-attracting chemokine; *Hb*, hemoglobin; *Lymph%*, proportion of lymphocytes; *PLR*, platelet-to-lymphocyte ratio; *LnSII*, natural logarithm of SII. Data are presented as mean ± standard deviation or median (interquartile range). Independent samples t-test was employed for *Prenatal Hb and LnSII*. Mann-Whitney U test was used for *IL-2rα*, *IL-9*, *TNF-β*, *MIP1β*, *CTACK*, *Lymph%* and PLR. * P <0.05; ** P <0.01; *** P <0.001; **** P <0.0001.

ELISA was employed to quantify IL2Rα, MIP1β, TNFβ, CTACK, and IL9 levels in maternal plasma obtained from a prospective cohort. Relevant predictive laboratory parameters (Prenatal Hb, Lymph%) were extracted from electronic medical records and hemogram-derived indices (PLR, LnSII) were calculated. The results revealed significant lower levels of IL2Rα (P<0.001), MIP1β (P<0.001), TNFβ (P<0.001), and IL9 (P<0.001) in the atonic PPH group compared to the control group. Conversely, CTACK levels were found to be significantly elevated in women with atonic PPH compared to the matched control group (P<0.001). Furthermore, lower levels of Prenatal Hb (P = 0.004), PLR (P = 0.007), and LnSII (P = 0.005) were observed in women diagnosed with atonic PPH, while Lymph% (P = 0.026) showed a significant increase in this group (**Table 4**, **Figure 7**).

**Table 4:** Validation of differentially expressed markers in a prospective cohort.

Parameters	Control N=41	Atonic PPH N=41	*P* value
IL-2Rα (pg/ml)	37.84 (32.26, 41.75)	23.23 (19.22, 31.92)	<0.001
IL-9 (pg/ml)	164.64 (154.65, 182.70)	122.29 (114.21, 130.68)	<0.001
TNFβ (pg/ml)	130.15 (121.21, 139.97)	109.26 (102.57, 116.36)	<0.001
MIP1β (pg/ml)	201.32 (180.83,223.09)	169.43 (157.86,185.98)	<0.001
CTACK (pg/ml)	103.98 (90.95,127.04)	147.65 (135.94,177.52)	<0.001
Prenatal Hb (g/l)	123.42 ± 10.46	117.95 ± 5.42	0.004
Lymph%	16.35 (13.21,19.43)	18.51 (15.39,20.63)	0.026
PLR	145.59 (124.26,182.05)	126.31 (100.53,151.22)	0.007
LnSII	6.94 ± 0.47	6.63 ± 0.47	0.005

Data are presented as mean ± standard deviation or median (interquartile range).

IL, interleukin; CTACK, cutaneous T cell-attracting chemokine; MIP-1β, macrophage inflammatory protein-1β; TNF-β, tumor necrosis factor-β; Hb, hemoglobin; Lymph%, lymphocyte ratio; PLR, platelet-to-lymphocyte ratio; SII, systemic immune-inflammation index; LnSII, natural logarithm of SII.

### Evaluation of models

#### Discrimination

**Figure 8:**
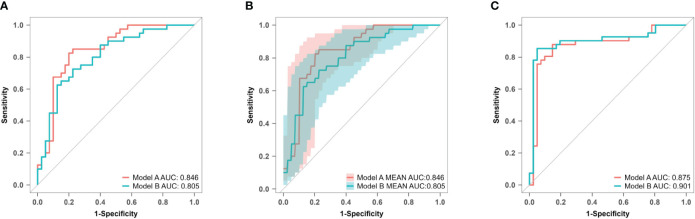
ROC curves of model A and model B for predicting atonic PPH. **(A)** ROC curves of predictive models A and B in the training set. Model A (AUC: 0.846, 95% CI 0.757–0.934, P< 0.001), model B (AUC: 0.805, 95% CI 0.709–0.901, *P*< 0.001). **(B)** ROC curves of predictive models A and B in the internal validation set. Model A (AUC: 0.876, 95% CI 0.749–0.930, P< 0.001), model B (AUC: 0.805, 95% CI 0.701–0.894, *P*< 0.001). **(C)** ROC curves of predictive models A and B in the temporal validation set. Model A (AUC: 0.875, 95% CI 0.789–0.961, P< 0.001), model B (AUC: 0.901, 95% CI 0.824–0.979, *P*< 0.001). *ROC* receiver operating characteristic, *AUC* area under the ROC curve.

The AUC values for model A in the training set, internal validation set, and temporal validation set were 0.846 (0.757–0.934), 0.846 (0.749–0.930), and 0.875 (0.789–0.961) respectively. The corresponding AUC values for model B were 0.805 (0.709–0.901), 0.805 (0.701–0.894), and 0.901 (0.824–0.979). ROC curves was plotted for both model A and model B (**Figure 8**). The accuracy, sensitivity, and specificity of model A in the training cohort were 0.813, 0.850, and 0.775 respectively, while in the validation cohort they were 0.756, 0.902 and 0.625 respectively. The corresponding values for model B in the training cohort were 0.750, 0.725 and 0.775, whereas in the validation cohort they were measured as 0.841, 0.854 and 0.829.

### Calibration

**Figure 9:**
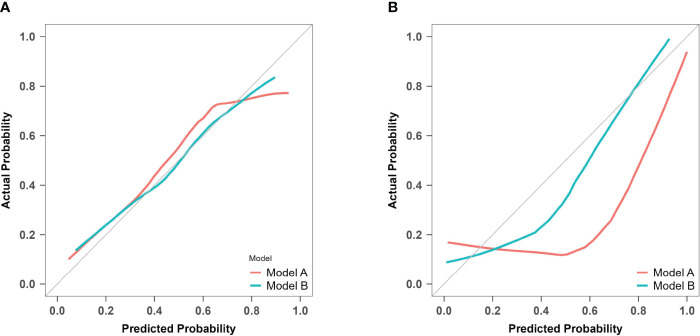
Calibration curves of model A and model B for predicting atonic PPH. **(A)** Calibration curves for the training cohort of predictive models A and B The red line (Bias corrected line for model A) and the green line (Bias corrected line for model B) represent the performance during internal validation by bootstrapping (*B* = 1000 repetitions). **(B)** Calibration curves for the prospective validation of predictive models A and B The red line (Apparent line for model A) and the green line (Apparent line for model B) represent the original performance in a prospective cohort.

For model A, the *P* values of the H-L test were 0.088 in the training set and< 0.001 in the prospective validation cohort. For model B, the corresponding values were 0.827 and 0.158. Calibration curves for both models are presented in **Figure 9**. The results indicate that model A exhibited poor fitting performance on the prospective validation set. Furthermore, the calibration curve revealed that the predicted values of model A demonstrated greater deviation from the ideal calibration curve. In contrast, model B exhibits superior calibration on the prospective validation set.

### Clinical utility

**Figure 10:**
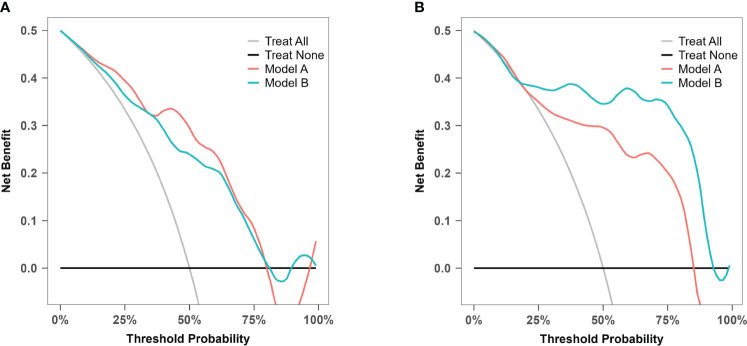
Decision curve analysis for model A and model B. **(A)** Decision curve analysis for prediction model A and B in the training cohort. **(B)** Decision curve analysis for prediction model A and B in the prospective validation cohort.

DCA was used to evaluate model A and model B in both the training set and the temporal validation set (**Figure 10**). Both models showed better net benefit for predicting atonic PPH across all potential thresholds (0% to 80%). This suggested that both models achieved a more optimal balance of clinical intervention for atonic PPH, regardless of the chosen risk threshold.

## Discussion

Using LASSO and multivariate logistic regression, we developed predictive models A and B for atonic PPH in “low-risk” women based on samples from a retrospective cohort and matched controls. The performance of the models was assessed through both internal and temporal validation. The findings of this study suggested that model B was promising for clinical implementation.

Early identification of risk factors allows obstetric healthcare professionals to adequately prepare for potentially life-threatening hemorrhages (1). Numerous relevant risk factors are currently used in predictive models, with almost all mentioned in the literature being clinical indicators. The variables frequently included in predictive models include parity, antenatal hemoglobin level, antepartum bleeding, maternal age ≥35 years old, gestational age, newborn weight, multiple pregnancies, obesity, a history of previous cesarean section, and placenta previa (18). For instance, the California Maternal Quality Care Collaborative (CMQCC) developed risk stratification tools for PPH based on major clinical and laboratory risk factors (19). A retrospective study involving 10,134 patients demonstrated that this tool correctly identified 80% of severe PPH cases, but its specificity was only 60% (3). Besides, limited research has been performed specifically focusing on PPH in low-risk populations without apparent risk factors related to PPH. The PPH in these women, however, does not meet the expected low levels. There is thus still an urgent need to identify highly effective novel biomarkers for predicting PPH.

Prior to delivery, immune cells accumulate in the maternal reproductive system and at maternal-fetal interface through cytokine secretion, establishing an inflammatory environment (20–22). These cytokines play a pivotal role in the initiation of labor and their dysregulation may contribute to PPH. Jiang et al. reported significantly elevated levels of basic FGF, IL1α, IL1β, IL1ra, IL2Rα, IL16, IL18, M-CSF, MIP1α, β-NGF, TRAIL and SCF were associated with an increased risk of atonic PPH (23). Gallo et al. found significantly higher concentrations of IL16, IL6, IL12/IL23p40, MCP1 and IL1β in the PPH group compared to the control group (24). Both studies identified significant biomarkers including IL16 and IL1β associated with PPH. In this study, we observed notable levels of IL2Rα, TNFβ, MIP1β, IL9 and higher level of CTACK in atonic PPH group. The discrepancy between our study results and previous studies (23, 24) may be attributed to differences in the populations included and the timing of specimen collection: specimens collected by Jiang et al. were obtained in late third trimester (37–41 weeks), while those collected by Gallo et al. were within three days prior delivery. However, the samples in this study were collected during the latent phase of the first stage of labor, characterized by the presence of regular contractions. Cytokine concentrations undergo continuous changes throughout pregnancy and labor progression. Further research is needed to investigate the correlation between cytokine levels at different gestational time points and their association with PPH.

Hemogram and coagulation parameters have significant predictive value for PPH. A previous case-control study showed that individuals with a fibrinogen level below 2 g/L are significantly predisposed to severe bleeding (25). Another retrospective study identified PLT< 150×10^9^/L and an increased APTT ratio as independent risk factors for PPH (26). SII has been proven to have prognostic and diagnostic value in various diseases including infection, tumor development, and cardiovascular disease, indicating its potential significance for assessing immune status and the inflammatory response (27–29). However, the relationship between SII and pregnancy-related outcomes remains unclear. Our findings suggest that prenatal Hb levels, Lymph% PLR, and LnSII showed certain predictive value for atonic PPH.

In contrast to the case for other pregnancy-related complications, the prediction of PPH is theoretically more accurate as parturition approaches. The present study explored the incidence of atonic PPH from a novel perspective, specifically investigating the correlation between maternal biomarkers and the incidence of PPH at the presence of regular uterine contraction. By using peripheral blood samples collected during the latent first stage after spontaneous labor, this study identified novel biomarkers for atonic PPH. Secondly, to address potential issues of multicollinearity compromising model robustness when solely using a logistic regression model, this study initially screened for factors showing intergroup differences and subsequently adding LASSO to logistic regression analysis. This study revealed low prenatal Hb, PLR, IL2Rα, and IL9 as factors independently predictive of atonic PPH. The predictive model based on these four factors exhibited favorable validation outcomes in a temporal validation cohort, emphasizing its clinical utility and suggesting its potential as a viable model for predicting atonic PPH.

The limitations of this study include a relatively small sample size and data derived from a single center, which may introduce selection bias. Future research should focus on larger sample sizes, multi-center data, and prospective trials to further identify and confirm risk factors for atonic PPH in the general population. This study did not include women who underwent cesarean section, since the onset of delivery is closely related to inflammatory responses and selective cesarean section is unrelated to this process. Additionally, owing to different immune characteristics between women undergoing spontaneous labor and induced labor (30), this study does not involve the population undergoing induced labor. The cohort data used in this study only include primiparous women because the labor characteristics of primiparous and multiparous women differ. Further studies should expand the demographic category. For both model A and model B, the AUC values obtained from prospective validation were higher than those from the training set, which may be attributable to changes in detection methods. In the training set, Bio-Plex Pro Human Cytokine 48-Plex Screening Panel was used to simultaneously determine the expression levels of 48 cytokines. However, targeted validation involving ELISA was performed on targeted cytokines in the prospective cohort. This study found that intergroup differences in cytokine levels detected by ELISA were more pronounced than those observed in the training set. In future research, our established predictive models should be validated using consistent testing methods.

## Conclusion

In conclusion, our research findings suggested that several plasma cytokines are dysregulated during the latent phase of labor in “low-risk” women who later develop atonic PPH. By using LASSO and logistic regression models, we were able to accurately assess the risk of atonic PPH in prospective cohorts. This approach allows early identification of high-risk individuals among pregnant women diagnosed with a “low risk” of bleeding, thereby significantly reducing the incidence and mortality rates associated with PPH in vaginal deliveries.

## Data availability statement

The original contributions presented in the study are included in the article/Supplementary Material, further inquiries can be directed to the corresponding author/s.

## Ethics statement

The studies involving humans were approved by Ethics Committee of Tianjin Central Hospital of Gynecology Obstetrics. The studies were conducted in accordance with the local legislation and institutional requirements. The participants provided their written informed consent to participate in this study.

## Author contributions

PZ: Formal Analysis, Methodology, Project administration, Validation, Writing – original draft. YJ: Conceptualization, Visualization, Writing – review & editing. HS: Investigation, Writing – review & editing. YF: Investigation, Writing – original draft. YL: Data curation, Resources, Software, Writing – review & editing. HG: Formal Analysis, Methodology, Writing – review & editing. YZ: Data curation, Visualization, Writing – review & editing. HC: Project administration, Supervision, Writing – review & editing. XC: Conceptualization, Funding acquisition, Resources, Supervision, Writing – review & editing.
