# Supplementary material for: Novel biomarkers for prediction of atonic postpartum hemorrhage among ‘low-risk’ women in labor
Source: Front Immunol. 2024 Jul 11;15:1416990. doi: 10.3389/fimmu.2024.1416990 (PMC11269088; doi:10.3389/fimmu.2024.1416990)
Supplement: Supplementary file 1 [file DataSheet_1.docx]

**Additional files**

**For**

**Novel biomarkers for prediction of atonic postpartum hemorrhage among ‘low-risk’ women in labor**

Pei Zhang^1,2,3^, Hui Song^1,2,3^, Yifan Fan^1,2,3^, Yanju Jia^2^, Yan Lv^2^, Hao Geng^1,2,3^, Ying Zhao^1,2,3^, Hongyan Cui ^1,2^, Xu Chen^1,2,3*^

***Additional file 1：Detailed methods (P1-P3)***

***Additional file 2：Supplementary tables and figures (P3-P11)***

***Additional file 3：TRIPOD checklist (P11-P14)***

**Additional file 1：Detailed methods**

# Relevant definitions according to WHO clinical guidelines

**Labor** is characterized by regular and progressively intensifying uterine contractions lasting 30 seconds or longer. These contractions occurred at intervals of 5-6 minutes, accompanied by progressive disappearance of cervical canal, dilatation of uterine orifice and descent of the fetus presentation spontaneously.

**The latent first stage** is a period of time characterized by regular uterine contractions and variable changes of the cervix, including some degree of effacement and slower progression of dilatation up to 5 cm.

**Postpartum hemorrhage (PPH)** is termed as excessive bleeding (≥500ml) in the first 24 hours post birth.

**Atonic Postpartum hemorrhage (Atonic PPH)** is diagnosed based on specific criteria: vaginal bleeding(≥500ml) in the first 24 hours, following spontaneous placental expulsion and identification of a flaccid uterus during physical examination. Exclusions included placental factors (such as all types of placenta previa detected via ultrasound and placental examination), cases with severe lacerations of the birth canal, and PPH resulting from coagulopathy.

**Subject recruitment**

We conducted a nested case-control study based on the hospitalized population-based cohort to identify plasma biomarkers of atonic PPH among low-risk primiparas in spontaneous labor. Based on our extensive biobank of a large cohort of pregnant women, we collected peripheral blood samples during the latent first stage of labor following spontaneous onset between April 2022 to November 2023.

Primiparas with full-term pregnancies were invited to participate in the cohort study on a voluntary basis. The inclusion criteria were as follows: primipara with full-term singleton infants, fetal head presentation and age range of 18 to 34 years. The following exclusion criteria were applied: (1) women with autoimmune diseases; (2) women with pregnancy comorbidities and complications potentially associated with PPH (including preeclampsia, poorly controlled or medically controlled gestational diabetes mellitus, placental abruption, scarred uterus, coagulopathy, and induced labor); (3) women who delivered low-birth-weight infants or macrosomia clinically diagnosed after birth; (4) PPH cases caused by placental factors (identified through ultrasound and placental examination); (5) traumatic PPH cases with severe lacerations of the birth canal; (6) women with intrauterine infection; (7) cases positive for group B streptococcus. Given the high prevalence of obstetric lacerations, it is worth noting that most minor ones do not result in significant PPH. Therefore, our study included individuals with mild obstetric lacerations, defined as mild cervical lacerations (＜1cm) or first-degree lacerations of perineum. Furthermore, these lacerations would be confirmed by a senior doctor and not considered as the primary cause of bleeding.

Subjects who experienced atonic PPH after vaginal delivery were assigned to the atonic PPH group, while the remaining pregnant women without PPH were matched to the control group in a 1:1 ratio using propensity scores. Gestational age, BMI (both before pregnancy and at delivery) and fetal birth weight were considered as predictors for PPH outcome during sample collection, and logistic regression was used to calculate the propensity score. Propensity matching was performed using maximized execution performance without replacement, with a caliper width set at 0.02 standard deviations of the logit of propensity score for the propensity score.

The baseline characteristics were extracted from the electronic medical record, while obstetric and perinatal complications data were collected through timely telephone follow-up by trained nurses.

**Sample collection**

Specimens were collected during the latent first stage after spontaneous labor. 6 ml peripheral blood were taken from each participant via venipuncture using EDTA anticoagulant tubes for detection. After centrifugation at 3000 rpm for 10 min at 4 °C, the supernatant plasma was separated and stored at -80 °C.

# Kit for cytokine profiling

Bio-Plex Pro Human Cytokine 48-Plex Screening Panel,1 x 96-well, includes coupled magnetic capture beads, premixed detection antibodies, standards, quality control, detection antibody diluent HB, standard diluent HB, sample diluent HB, assay buffer, 10x wash buffer, streptavidin-PE, 96-well flat bottom plate, sealing tape, and instructions, for the detection of basic FGF, CTACK, eotaxin, G-CSF, GM-CSF, GRO-α, HGF, IFN-α2, IFN-γ, IL-1α, IL-1β,IL-1ra, IL-2, IL-2Rα, IL-3, IL-4, IL-5, IL-6, IL-7, IL-8, IL-9, IL-10, IL-12 (p40), IL-12 (p7O), IL-13, IL-15, IL-16, IL-17A, IL-18, IP-10, LIF, MCP-1 (MCAF),MCP-3, M-CSF, MIF, MIG, MIP-1α, MIP-1β, β-NGF, PDGF-BB, RANTES, SCF,SCGF-β, SDF-1α, TNF-α, TNF-β, TRAIL, VEGF-A, VCAM-1. The levels of IL3, VEGF-A, IL5, and IL-15 were undetectable in the majority of samples in this study, thus precluding their comparison.

# Assessment of potential biomarkers

## Cytokines profiling

The frozen-thawed plasma was collected and diluted 2-fold with a diluent. Subsequently, 50 uL sample was taken for testing. The Bio-Plex Pro Human Cytokine Screening 48-Plex Panel (Bio-Rad, #12007283), which integrates a network of biologically relevant cytokines andchemokines in a single assay. Polystyrene microspheres were stained with different fluorescent colors using varying ratios of two red sorting fluorescent dyes, resulting in microspheres encoded with up to 100 fluorescences. The target antibody molecules against different analytes were covalently cross-linked to specific coding microspheres, where each coding microsphere corresponded to the corresponding detection item. The fluorescent coding microspheres were mixed with different analytes, and subsequently the complexes formed were combined with the labeled fluorescein. The microspheres were propelled by a flowing sheath solution in a single row and passed through the laser sequentially to determine the fluorescence intensity of the reporter molecule. The instrument read the fluorescence value of the sample and substituted it into a fitting curve to calculate concentrations. Data analysis was performed using MILLIPLEX Analyst. Version 5.1. Representative assay working ranges, assay sensitivity, and precision were presented in sTable 1.

## Routine laboratory tests

Results of the last laboratory test before delivery, including blood cell analysis and coagulation tests, were obtained on patients’ admission. The complete blood count parameters were analyzed using the Cell-Dyn 3700 (Abbott Diagnostics, Santa Clara, CA, USA). Coagulation tests were conducted using the Sysmex CS 2500 System coagulation analyzer (Siemens Healthcare Diagnostics, Erlangen, Germany). All measurements were performed within a two-hour time frame after blood sampling. Neutrophil-to-lymphocyte ratio (NLR), monocyte-to-lymphocyte ratio (MLR), platelet-to-lymphocyte ratio (PLR), systemic immune-inflammation index (SII) and LnSII, which were reported to be related to systemic immune response previously, were calculated based on these laboratory test parameters. The SII was obtained by multiplying the neutrophil count with the PLR and LnSII was the natural logarithm of SII.

## Enzyme-linked immunosorbent assay (ELISA)

ELISA were used to verify the concentrations of differential cytokines in the prospective cohort. Human IL-2Rα, TNF-β, MIP-1β, IL-9, and CTACK ELISA kits were procured from Thermo Fisher Scientific (Waltham, MA, USA). Plasma samples were appropriately diluted with manufacturer-provided diluents to ensure absorbance readings fell within the range of the standard curve. The concentration of each sample was determined by constructing a linear standard curve using the software provided with the microplate reader as per the manufacturer's instructions.

**Additional file 2：Supplementary tables and figures**

# Table S1 Representative performance characteristics

|  | **Assay Working Range** | | **Assay Sensitivity** |  |  |  |
| --- | --- | --- | --- | --- | --- | --- |
|  | **pg /ml** | | **pg /ml** | **Mean Intra-Assay** | **Mean Inter-Assay** | **Single plex Bead** |
| **Analyte** | **LLOQ*** | **ULOQ*** | **LOD*** | **%CV*** | **%CV*** | **Region** |
| Basic FGF | 3.26 | 3341 | 2.54 | 3.1 | 2.4 | 44 |
| CTACK | 2.10 | 15656 | 0.82 | 2.7 | 5.2 | 72 |
| Eotaxin | 0.14 | 2281 | 0.05 | 4.4 | 1.2 | 43 |
| G-CSF | 6.35 | 104106 | 3.63 | 3.1 | 4.0 | 57 |
| GM-CSF | 0.48 | 7846 | 0.19 | 4.3 | 2.2 | 34 |
| GRO-α | 21.05 | 31255 | 13.45 | 2.6 | 7.9 | 61 |
| HGF | 8.76 | 143513 | 7.09 | 2.6 | 2.9 | 62 |
| IFN-α2 | 0.95 | 15569 | 0.46 | 3.3 | 4.4 | 20 |
| IFN-γ | 1.57 | 25665 | 1.05 | 3.1 | 3.6 | 21 |
| IL-1α | 3.73 | 61154 | 6.65 | 3.5 | 4.9 | 63 |
| IL-1β | 0.29 | 4672 | 0.24 | 3.6 | 3.2 | 39 |
| IL-1ra | 6.21 | 34949 | 3.16 | 4.7 | 5.1 | 25 |
| IL-2 | 1.29 | 21178 | 0.75 | 1.7 | 2.5 | 38 |
| IL-2Ra | 1.48 | 24270 | 1.65 | 3.4 | 4.8 | 13 |
| IL-3 | 0.13 | 2139 | 0.13 | 5.0 | 3.9 | 64 |
| IL-4 | 0.19 | 3064 | 0.09 | 3.2 | 1.9 | 52 |
| IL-5 | 3.63 | 59499 | 0.86 | 2.3 | 2.3 | 33 |
| IL-6 | 0.38 | 6244 | 0.34 | 2.2 | 3.0 | 19 |
| IL-7 | 1.92 | 31475 | 1.22 | 2.7 | 3.9 | 74 |
| IL-8 | 0.85 | 13992 | 0.36 | 3.2 | 2.8 | 54 |
| IL-9 | 3.62 | 31527 | 1.08 | 2.6 | 7.1 | 77 |
| IL-10 | 1.06 | 17427 | 0.69 | 2.3 | 3.4 | 56 |
| IL-12(p40) | 14.68 | 240582 | 6.39 | 4.5 | 2.4 | 28 |
| IL-12(p70) | 1.43 | 23425 | 0.78 | 3.3 | 2.9 | 75 |
| IL-13 | 0.31 | 5157 | 0.22 | 3.1 | 2.7 | 51 |
| IL-15 | 12.42 | 203426 | 12.82 | 2.8 | 4.1 | 73 |
| IL-16 | 1.20 | 19639 | 0.25 | 2.5 | 3.0 | 27 |
| IL-17 | 2.44 | 39972 | 1.16 | 2.4 | 1.4 | 76 |
| IL-18 | 0.66 | 10892 | 0.31 | 2.9 | 2.2 | 42 |
| IP-10 | 3.41 | 34953 | 1.43 | 2.8 | 6.0 | 48 |
| LIF | 3.86 | 53806 | 2.05 | 2.5 | 4.7 | 29 |
| MCP-1 (MCAF) | 0.53 | 8755 | 0.44 | 3.2 | 3.4 | 53 |
| MCP-3 | 0.48 | 4899 | 0.24 | 4.4 | 4.2 | 26 |
| M-CSF | 0.75 | 12290 | 0.27 | 2.4 | 3.6 | 67 |
| MIF | 2.70 | 44168 | 2.45 | 3.4 | 4.7 | 35 |
| MIG | 3.16 | 32365 | 1.39 | 4.4 | 4.2 | 14 |
| MIP-1a | 0.12 | 1218 | 0.06 | 4.5 | 4.2 | 55 |
| MIP-1β | 1.41 | 1439 | 1.41 | 3.4 | 2.5 | 18 |
| β-NGF | 0.47 | 7655 | 0.23 | 2.9 | 3.9 | 46 |
| PDGF-BB | 7.12 | 37133 | 2.96 | 3.3 | 9.7 | 47 |
| RANTES | 16.72 | 26467 | 3.98 | 3.0 | 6.7 | 37 |
| SCF | 1.82 | 29899 | 0.99 | 4.1 | 2.6 | 65 |
| SCGF-β | 82.11 | 1345200 | 141.77 | 2.3 | 3.8 | 78 |
| TNF-α | 3.33 | 54566 | 1.13 | 3.5 | 3.0 | 36 |
| TNF-β | 0.80 | 13186 | 0.38 | 3.0 | 4.7 | 30 |
| TRAIL | 1.78 | 29188 | 0.89 | 3.2 | 4.5 | 66 |
| VEGF-A | 18.01 | 149830 | 10.16 | 2.8 | 8.5 | 45 |

* The LLOQ, ULOQ, LOD, and inter-assay precision %CV are mean data determined from three independent multiplex assays in a serum-based matrix. Intra-assay %CV is derived from one representative assay. LLOQ and ULOQ are defined as the boundary standard curve points within which the performance specifications of individual standard points were met for a 10% intra-assay CV and recovery range of 70-130%. Data were generated using the magnetic workflow with the Bio-Plex Pro Wash Station.

Basic FGF, basic fibroblast growth factor. CTACK cutaneous T cell-attracting chemokine eotaxin. G-CSF, granulocyte-colony stimulating factor. GM-CSF, granulocyte-macrophage colony stimulating factor. GRO-α, growth-regulated oncogene-α. HGF, hepatocyte growth factor. IFN-α2, interferon-α2. IFN-γ, interferon-γ. IL, interleukin. IP-10, interferon-inducible protein-10. LIF, leukemia inhibitory factor. MCP-1, monocyte chemotactic protein-1. MCP-3, monocyte chemotactic protein-3. M-CSF, macrophage colony stimulating factor. MIF, migration inhibitor factor. MIG, monokine induced by IFN-γ. MIP-1α, macrophage inflammatory protein-1α. MIP-1β, macrophage inflammatory protein-1β. β-NGF, β-nerve growth factor. PDGF-BB, platelet-derived growth factor-BB. RANTES, regulate upon activation normal T cell expressed and secreted. SCF, stem cell factor. SCGF-β, stem cell growth factor-β. SDF-1α, stromal cell-derived factor-α. TNF-α, tumor necrosis factor-α. TNF-β, tumor necrosis factor-β. TRAIL, tumor necrosis factor-related apoptosis-induced ligand. VEGF-A, vascular endothelial growth factor A. VCAM-1, vascular cell adhesion molecule-1.

# Table S2 ROC analysis of the predictive value of 9 biomarkers for atonic PPH

| Predictors | Cut Off | AUC | AUC.SE | AUC.Pvalue |
| --- | --- | --- | --- | --- |
| Prenatal Hb(g/L) | 116.500 | 0.637(0.514,0.760) | 0.063 | 0.017 |
| Lymph% | 18.300 | 0.688(0.568,0.807) | 0.061 | 0.002 |
| PLR | 168.554 | 0.641(0.519,0.762) | 0.062 | 0.015 |
| lnSII | 6.863 | 0.705(0.589,0.821) | 0.059 | 0.001 |
| IL2Rα(pg/ml) | 21.505 | 0.715(0.600,0.829) | 0.058 | <0.001 |
| MIP1β(pg/ml) | 195.960 | 0.626(0.501,0.752) | 0.064 | 0.026 |
| TNFβ(pg/ml) | 158.265 | 0.657(0.535,0.780) | 0.063 | 0.008 |
| CTACK(pg/ml) | 67.515 | 0.638(0.513,0.764) | 0.064 | 0.017 |
| IL9(pg/ml) | 156.405 | 0.677(0.554,0.800) | 0.063 | 0.003 |

** P <0.05; ** P <0.01; *** P <0.001.*

*IL*, interleukin. *CTACK*, cutaneous T cell-attracting chemokine eotaxin, *MIP-1β* macrophage inflammatory protein-1β, *TNF-β* tumor necrosis factor-β, *Hb* hemoglobin, *Lymph%* lymphocyte ratio, *PLR* platelet to lymphocyte ratio, *SII* systemic immune-inflammation index, *LnSII* the natural logarithm of SII, *AUC* area under the curve, *SE* standard error.

# Table S3 The prediction model A with multivariate logistic regression

| Intercept and variables | β | OR | SE | Statistic | 95% CI | P value |
| --- | --- | --- | --- | --- | --- | --- |
| Intercept | 19.829 | 4.087e+08 | 12.866 | 1.541 | (0.010, 2.020) | 0.123 |
| IL2Rα | -0.071 | 0.932 | 0.032 | -2.172 | 0.870, 0.990 | 0.030 |
| MIP1β | -0.017 | 0.983 | 0.019 | -0.926 | 0.946, 1.019 | 0.355 |
| TNFβ | -0.001 | 0.999 | 0.019 | -0.064 | 0.962, 1.037 | 0.949 |
| CTACK | 0.017 | 1.018 | 0.011 | 1.637 | 0.998, 1.041 | 0.102 |
| IL9 | -0.026 | 0.974 | 0.019 | -1.364 | 0.938, 1.012 | 0.172 |
| Prenatal Hb | -0.072 | 0.931 | 0.029 | -2.433 | (0.873, 0.982) | 0.015 |
| Lymph% | 0.054 | 1.056 | 0.105 | 0.520 | 0.864, 1.313 | 0.603 |
| PLR | -0.006 | 0.994 | 0.013 | -0.489 | 0.968, 1.016 | 0.625 |
| LnSII | -0.403 | 0.668 | 1.65 | -0.245 | 0.026, 18.301 | 0.807 |

Model A

$$\log\left( \frac{p}{1-p} \right)=19.829-0.072\times\mathbf{Prenatal Hb+}0.054\times\boldsymbol{Lymph\%}-0.006\times\mathbf{PLR}-0.403\times\mathbf{lnSII}-0.071\times\boldsymbol{IL2R\alpha}-0.017\times\boldsymbol{MIP1\beta}-0.001\times\boldsymbol{TNF\beta+}0.017\times\mathbf{CTACK}-0.026\times\mathbf{IL9}$$

*IL2Rα* interleukin-2 receptor subunit α, *MIP1β* macrophage inflammatory protein-1β, *TNF-β* tumor necrosis factor-β, *IL9* interleukin-9, *CTACK* cutaneous T cell-attracting chemokine, *Hb* hemoglobin, *Lymph%* lymphocyte ratio, *PLR* platelet to lymphocyte ratio, *SII* systemic immune-inflammation index, *LnSII* the natural logarithm of SII, *OR* odds ratio, *SE* standard error, *CI* confidence interval.

# Table S4 The prediction model B with multivariate logistic regression

| Intercept and variables | β | OR | SE | Statistic | 95% CI | *P* value |
| --- | --- | --- | --- | --- | --- | --- |
| Intercept | 18.986 | 1.760 | 5.369 | 3.537 | 1.436 2.546 | <0.001 |
| IL2Rα | -0.069 | 0.934 | 0.030 | -2.307 | 0.876, 0.986 | 0.021 |
| IL9 | -0.025 | 0.966 | 0.014 | -2.516 | 0.938, 0.991 | 0.012 |
| Prenatal Hb | -0.078 | 0.925 | 0.028 | -2.842 | 0.871, 0.972 | 0.004 |
| PLR | -0.014 | 0.986 | 0.007 | -2.164 | 0.972, 0.998 | 0.030 |

Model B

$$\log\left( \frac{p}{1-p} \right)=18.986-0.078\times\mathbf{Prenatal Hb}-0.014\times\mathbf{PLR}-0.069\times\boldsymbol{IL2R\alpha}-0.025\times\mathbf{IL9}$$

*IL2Rα* interleukin-2 receptor subunit α, *IL9* interleukin-9, *Hb* hemoglobin, *PLR* platelet to lymphocyte ratio, *OR* odds ratio, *SE* standard error, *CI* confidence interval.

# Table S5 The discrimination and calibration of prediction model A and B in the training cohort

| Model | Cut Off | AUC | AUC.SE | AUC.Pvalue | H-L.κ^2^ | H-L. P value |
| --- | --- | --- | --- | --- | --- | --- |
| Model A | 0.431 | 0.846(0.757,0.934) | 0.045 | <0.001 | 13.757 | 0.088 |
| Model B | 0.516 | 0.805(0.709,0.901) | 0.049 | <0.001 | 4.323 | 0.827 |

AUC area under the ROC curve, *SE* standard error, *H-L* Hosmer-Lemeshow test.

# Table S6 The discrimination of prediction model A and B in the internal validation cohort

| Model | AUC | AUC.SE | AUC. P value |
| --- | --- | --- | --- |
| Model A | 0.876(0.749,0.930) | 0.046 | <0.001 |
| Model B | 0.805(0.701,0.894) | 0.050 | <0.001 |

AUC area under the ROC curve, *SE* standard error.

# Table S7 The discrimination of prediction model A and B in the temporal validation cohort

| Model | Cut Off | AUC | AUC.SE | AUC.Pvalue | H-L.κ^2^ | H-L.Pvalue |
| --- | --- | --- | --- | --- | --- | --- |
| Model A | 0.811 | 0.875(0.789,0.961) | 0.044 | <0.001 | 66.817 | <0.001 |
| Model B | 0.582 | 0.901(0.824,0.979) | 0.040 | <0.001 | 11.855 | 0.158 |

AUC area under the ROC curve, *SE* standard error, *H-L* Hosmer-Lemeshow test.

# Figure S1


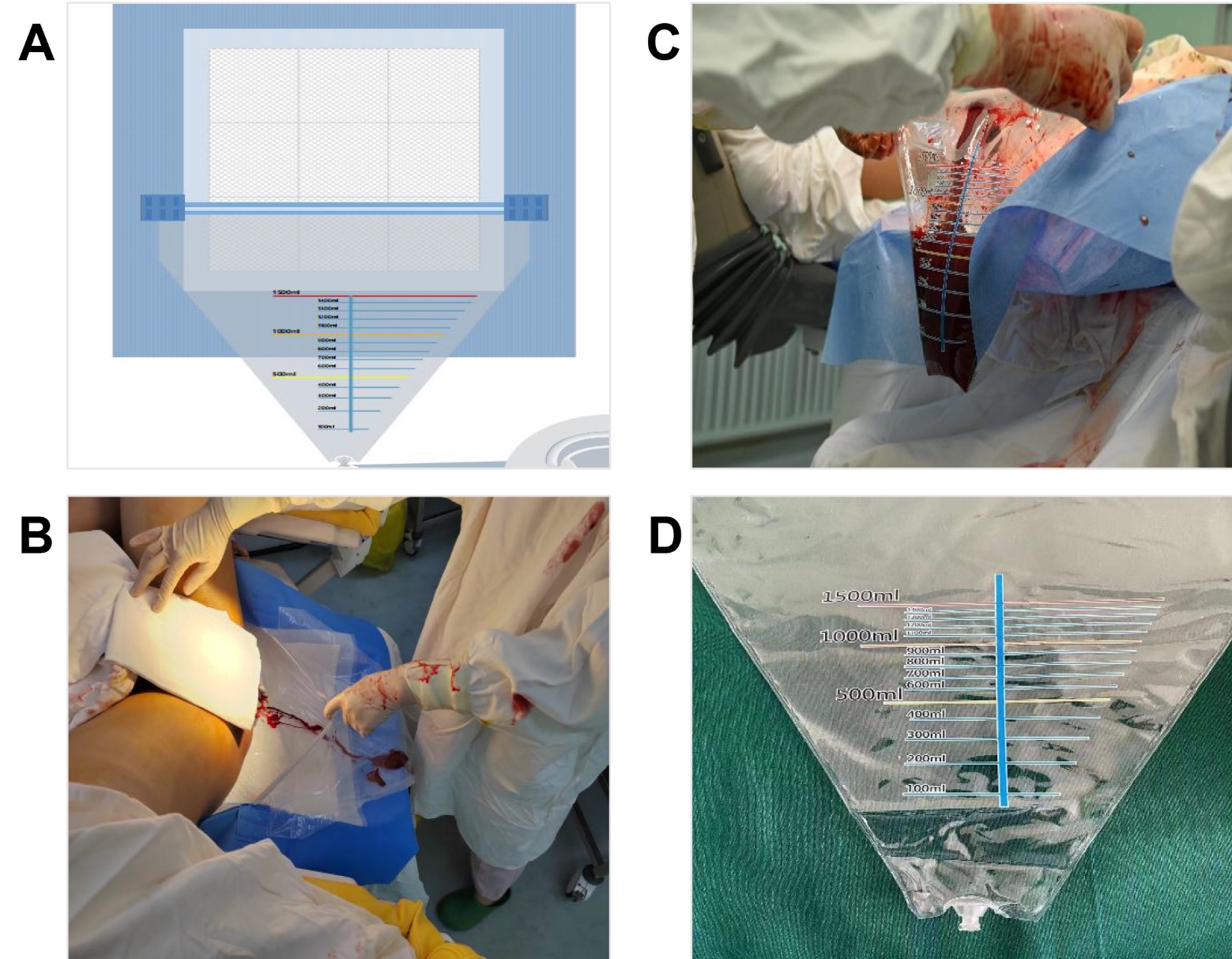


**Figure S1** Quantitative evaluation of blood loss. **(A)** Illustration of a blood collection bag model. (**B)** A case of PPH≥500 ml. (**C)** The usage instructions for the blood collection bag. **(D)** The V-shaped body is equipped with scale lines and warning lines, accompanied by a drainage port at the bottom. Intrapartum blood loss was measured utilizing a V-shaped blood collection bag with scale lines and warning lines. After the baby delivered and amniotic fluid was drained as much possible, the collection bag was positioned beneath the buttocks immediately until bleeding stopped. Upon the women’ return to the ward, the blood loss was quantified via the traditional weighing method (wet weight-dry weight of the bloody mat). The total volume of bleeding was calculated as the sum of the losses measured in both steps during 24 hours postpartum.

# Figure S2


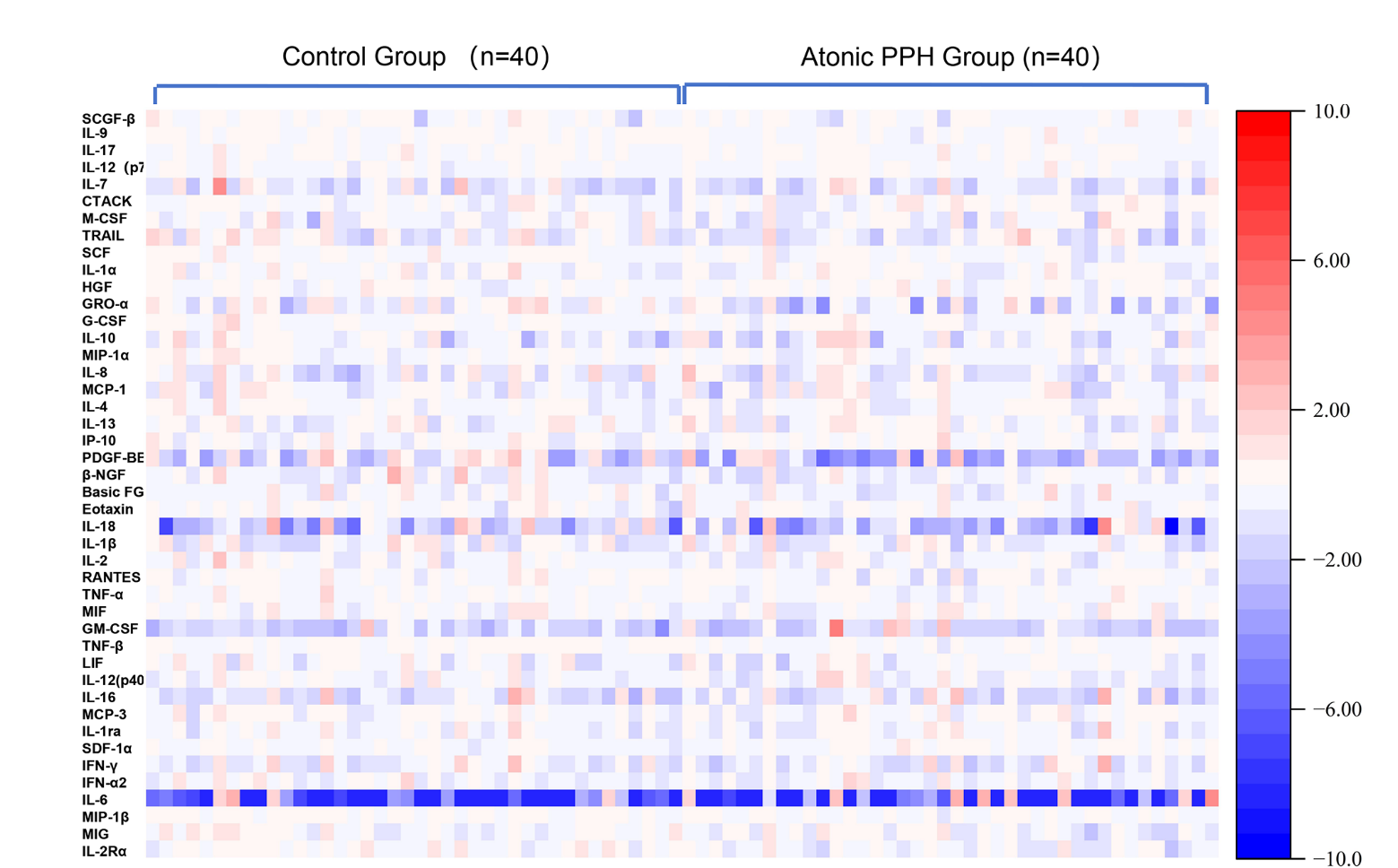


**Figure S2** Heat map of 48 cytokine concentrations for all participants in training cohort (40 in atonic PPH group and 40 in control group). Colors represent high (red) or low (blue) concentration.

# Figure S3


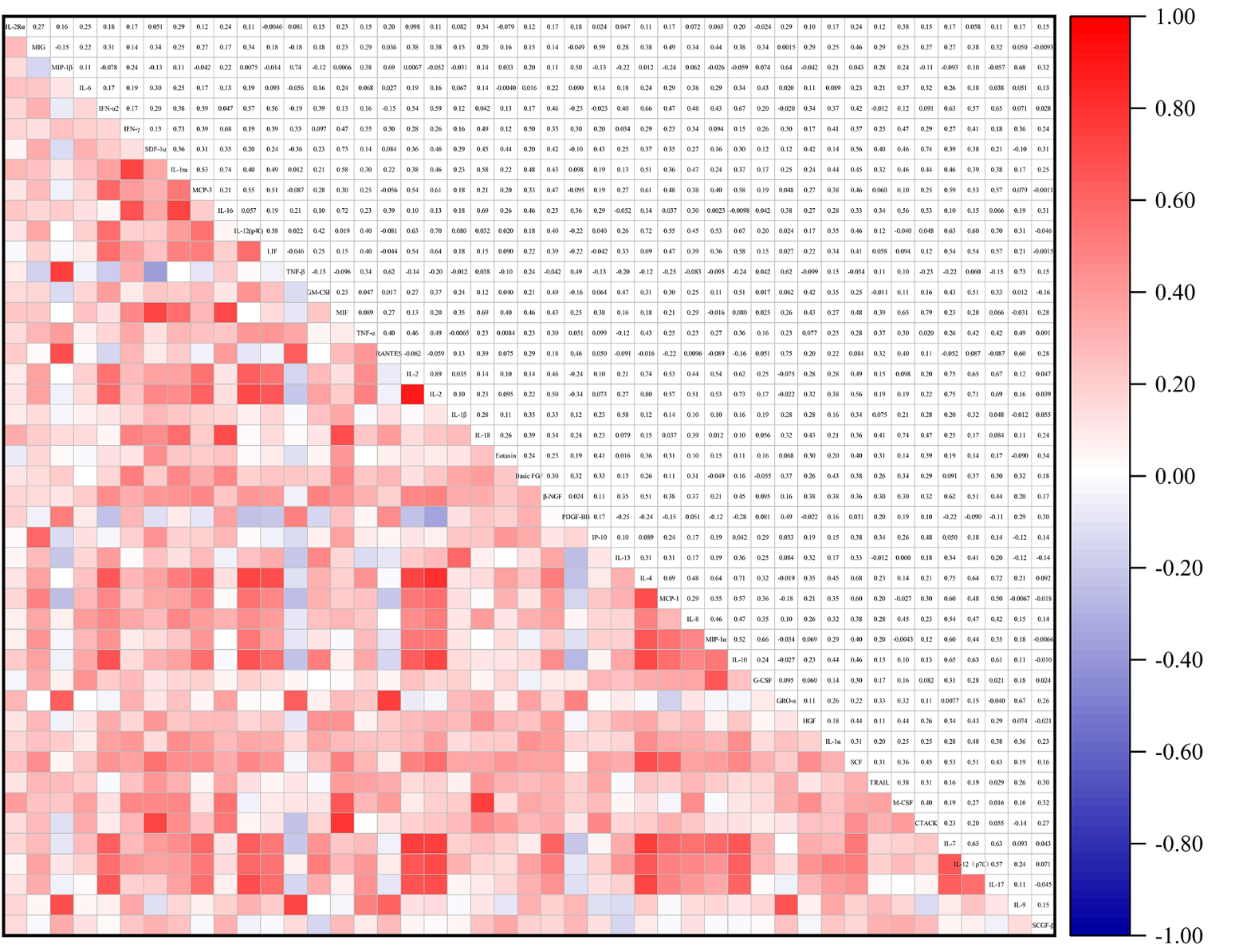


**Figure S3** Pairwise Pearson’s correlations among log-concentrations of 48 cytokines levels from training cohort (40 in atonic PPH group and 40 in control group). Colors represent high (red) or low (blue) relative expression levels.

#
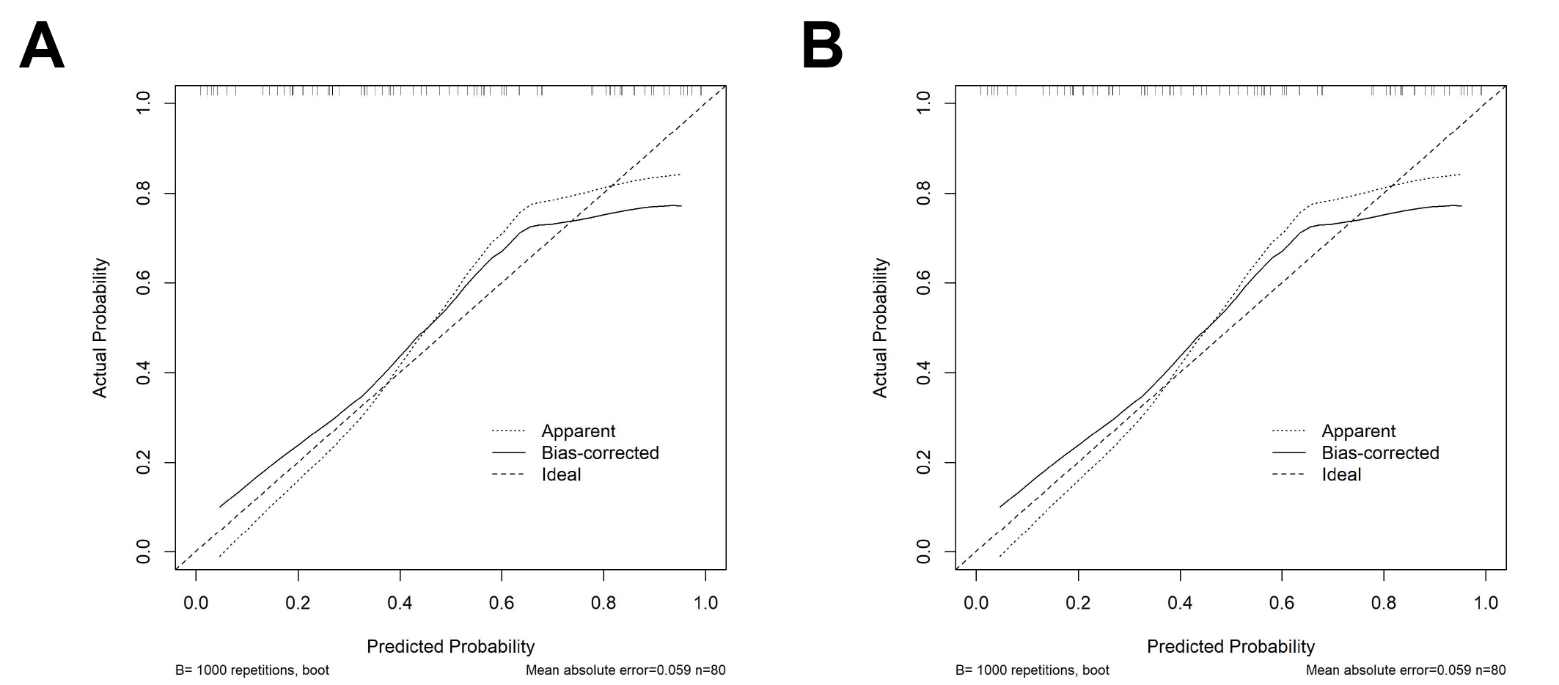
Figure S4

**Figure S4** Plot showing the comparison between the expected and observed incidence of atonic PPH in model A **(A)** and model B **(B)**, following apparent and bias-corrected calibration in the training cohort. The dashed line represents the original performance, and the solid dashed line represents the performance during internal validation by bootstrapping (*B* = 1000 repetitions). Results of the Hosmer-Lemeshow test demonstrate that the P-value was 0.088 for model A, and 0.827 for model B.

**Additional file 3：TRIPOD checklist**

This document contains a completed TRIPOD checklist for the manuscript: “Development and temporal validation of prediction models for atonic postpartum hemorrhage utilizing novel biomarkers among women in labor”.

Pei Zhang, Hui Song, Yifan Fan, Yanju Jia, Yan Lv, Hao Geng, Ying Zhao, Hongyan Cui and Xu Chen

Sections in the submitted manuscript are provided. For items that are not relevant in this study a “NA” has been written.

| **1Section/Topic** | **Item** |  | **Checklist Item** | **Sections** |
| --- | --- | --- | --- | --- |
| **Title and abstract** | | | | |
| Title | 1 | D;V | Identify the study as developing and/or validating a multivariable prediction model, the target population, and the outcome to be predicted. | Title |
| Abstract | 2 | D;V | Provide a summary of objectives, study design, setting, participants, sample size, predictors, outcome, statistical analysis, results, and conclusions. | Abstract |
| **Introduction** | | | | |
| Background and objectives | 3a | D;V | Explain the medical context (including whether diagnostic or prognostic) and rationale for developing or validating the multivariable prediction model, including references to existing models. | Intro para 1-4 |
|  | 3b | D;V | Specify the objectives, including whether the study describes the development or validation of the model or both. | Intro para 4 |
| **Methods** | | | | |
| Source of data | 4a | D;V | Describe the study design or source of data (e.g., randomized trial, cohort, or registry data), separately for the development and validation data sets, if applicable. | Methods para 1-2 |
|  | 4b | D;V | Specify the key study dates, including start of accrual; end of accrual; and, if applicable, end of follow-up. | Methods para 1 |
| Participants | 5a | D;V | Specify key elements of the study setting (e.g., primary care, secondary care, general population) including number and location of centres. | Methods para 1-2 |
|  | 5b | D;V | Describe eligibility criteria for participants. | Methods para 1 |
|  | 5c | D;V | Give details of treatments received, if relevant. | NA |
| Outcome | 6a | D;V | Clearly define the outcome that is predicted by the prediction model, including how and when assessed. | Methods para 1 |
|  | 6b | D;V | Report any actions to blind assessment of the outcome to be predicted. | NA |
| Predictors | 7a | D;V | Clearly define all predictors used in developing or validating the multivariable prediction model, including how and when they were measured. | Methods para 4-6 |
|  | 7b | D;V | Report any actions to blind assessment of predictors for the outcome and other predictors. | NA |
| Sample size | 8 | D;V | Explain how the study size was arrived at. | NA |
| Missing data | 9 | D;V | Describe how missing data were handled (e.g., complete-case analysis, single imputation, multiple imputation) with details of any imputation method. | Methods para 8 |
| Statistical analysis methods | 10a | D | Describe how predictors were handled in the analyses. | Methods para 7,  Figure 3 |
|  | 10b | D | Specify type of model, all model-building procedures (including any predictor selection), and method for internal validation. | Methods para 8 |
|  | 10c | V | For validation, describe how the predictions were calculated. | Methods para 8 |
|  | 10d | D;V | Specify all measures used to assess model performance and, if relevant, to compare multiple models. | Methods para 8 |
|  | 10e | V | Describe any model updating (e.g., recalibration) arising from the validation, if done. | NA |
| Risk groups | 11 | D;V | Provide details on how risk groups were created, if done. | NA |
| Development vs. validation | 12 | V | For validation, identify any differences from the development data in setting, eligibility criteria, outcome, and predictors. | NA |
| **Results** | | | | |
| Participants | 13a | D;V | Describe the flow of participants through the study, including the number of participants with and without the outcome and, if applicable, a summary of the follow-up time. A diagram may be helpful. | Result para 1,  Figure 1 |
|  | 13b | D;V | Describe the characteristics of the participants (basic demographics, clinical features, available predictors), including the number of participants with missing data for predictors and outcome. | Result para 1, Table 1 |
|  | 13c | V | For validation, show a comparison with the development data of the distribution of important variables (demographics, predictors and outcome). | Result para 5, Table 1 |
| Model development | 14a | D | Specify the number of participants and outcome events in each analysis. | Table 1 |
|  | 14b | D | If done, report the unadjusted association between each candidate predictor and outcome. | NA |
| Model specification | 15a | D | Present the full prediction model to allow predictions for individuals (i.e., all regression coefficients, and model intercept or baseline survival at a given time point). | Result para 5,  Table 2 and 3 |
|  | 15b | D | Explain how to the use the prediction model. | Result para 6 |
| Model performance | 16 | D;V | Report performance measures (with CIs) for the prediction model. | Result para 9, Figure 7 |
| Model-updating | 17 | V | If done, report the results from any model updating (i.e., model specification, model performance). | NA |
| **Discussion** | | | | |
| Limitations | 18 | D;V | Discuss any limitations of the study (such as nonrepresentative sample, few events per predictor, missing data). | Discussion para 6 |
| Interpretation | 19a | V | For validation, discuss the results with reference to performance in the development data, and any other validation data. | NA |
|  | 19b | D;V | Give an overall interpretation of the results, considering objectives, limitations, results from similar studies, and other relevant evidence. | Discussion para 1-4, 6 |
| Implications | 20 | D;V | Discuss the potential clinical use of the model and implications for future research. | Discussion para 5 |
| **Other information** | | | | |
| Supplementary information | 21 | D;V | Provide information about the availability of supplementary resources, such as study protocol, Web calculator, and data sets. | Supplementary data |
| Funding | 22 | D;V | Give the source of funding and the role of the funders for the present study. | Acknowledgement para |

*Items relevant only to the development of a prediction model are denoted by D, items relating solely to a validation of a prediction model are denoted by V, and items relating to both are denoted D;V. We recommend using the TRIPOD Checklist in conjunction with the TRIPOD Explanation and Elaboration document.
